# Modeling the Progression of Placental Transport from Early‐ to Late‐Stage Pregnancy by Tuning Trophoblast Differentiation and Vascularization

**DOI:** 10.1002/adhm.202301428

**Published:** 2023-11-07

**Authors:** Sonya Kouthouridis, Alexander Sotra, Zaim Khan, Justin Alvarado, Sandeep Raha, Boyang Zhang

**Affiliations:** ^1^ Department of Chemical Engineering McMaster University Hamilton ON L8S 4L8 Canada; ^2^ School of Biomedical Engineering McMaster University Hamilton ON L8S 4L8 Canada; ^3^ Department of Biochemistry and Biomedical Sciences McMaster University Hamilton ON L8S 4L8 Canada; ^4^ Department of Pediatrics and the Graduate Programme in Medical Sciences McMaster University Hamilton ON L8S 4L8 Canada

**Keywords:** differentiation, organs‐on‐a‐chip, perfusion, placenta, stem cells, syncytiotrophoblasts, vasculatures

## Abstract

The early‐stage placental barrier is characterized by a lack of fetal circulation and by a thick trophoblastic barrier, whereas the later‐stage placenta consists of vascularized chorionic villi encased in a thin, differentiated trophoblast layer, ideal for nutrient transport. In this work, predictive models of early‐ and late‐stage placental transport are created using blastocyst‐derived placental stem cells (PSCs) by modulating PSC differentiation and model vascularization. PSC differentiation results in a thinner, fused trophoblast layer, as well as an increase in human chorionic gonadotropin secretion, barrier permeability, and secretion of certain inflammatory cytokines, which are consistent with in vivo findings. Further, gene expression confirms this shift toward a differentiated trophoblast subtype. Vascularization results in a molecule type‐ and size‐dependent change in dextran and insulin permeability. These results demonstrate that trophoblast differentiation and vascularization have critical effects on placental barrier permeability and that this model can be used as a predictive measure to assess fetal toxicity of xenobiotic substances at different stages of pregnancy.

## Graphical abstract

Early‐ and late‐stage placental barrier models are developed using placental stem cells cultured in the perfusable IFlowPlate384 device and show differential barrier functionality, highlighting the importance of trophoblast differentiation and vascularization in placental modeling. Modeling different stages of pregnancy can therefore better inform drug safety standards for pregnant women.

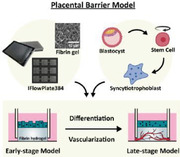

## 1 Introduction

The placenta is a transient organ which develops from both maternal and fetal cells during pregnancy and is expelled during parturition. It is indisputably the most important organ during pregnancy because it is responsible for feto‐maternal transport of nutrients, gases, and waste products, in addition to the secretion of important hormones responsible for pregnancy maintenance.^[^ ^1^ ^]^ However, its impermanence combined with the lack of good animal models which accurately represent human placentation is arguably the reason why it is so poorly understood. However, the search for better models that recapitulate the functions of the human placenta is driven by the observation that pregnant women have a greater susceptibility to infection and disease combined with the vulnerability of the developing fetus.^[^ ^2^ ^]^ This gap in knowledge makes it challenging to evaluate the risk and safety of drugs for use during pregnancy.

The study of the placenta is mostly limited to either tissue explants, from terminated or term placentas, and animal models. Although placental explants have provided invaluable insights on placental morphogenesis and dysfunction, explants have limited viability once removed from the body and require constant sourcing, which is often more difficult for first and second trimester placentas. In contrast, rodent models allow for controlled maternal exposure to various substances and conditions. However, there are significant functional and morphological differences between human and rodent placentas which impair the translation of animal study findings to clinical research.^[^ ^3^ ^]^ Human‐based in vitro placental models have recently been developed with the goal of modeling placental barrier function.^[^ ^4^, ^5^, ^6^, ^7^ ^]^ Placenta‐on‐chip cultures have been designed with trophoblast and endothelial cells cultured on opposites sides of either a semipermeable membrane or hydrogel,^[^ ^8^ ^]^ whose compartmentalized culture allows for simple molecular transport studies. The cultures are subjected to perfusion on each side: trophoblast channel perfusion to model maternal blood flow and endothelial channel perfusion to mimic fetal blood flow.^[^ ^9^, ^10^ ^]^ However, these models do not incorporate the branched morphology reminiscent of fetal vasculature within the placenta. Further, they often utilize trophoblast cell lines which are susceptible to genetic drift over time, further distancing them from the primary cells they are meant to represent.^[^ ^11^ ^]^

The choriocarcinoma BeWo cell line has been used in many of these barrier models,^[^ ^6^, ^9^, ^12^, ^13^ ^]^ despite the fact that cancer cell lines generally retain most of the genetic properties of their cancer of origin.^[^ ^14^ ^]^ BeWo cells exhibit low fusion capacities when grown to confluence and are therefore not representative of the healthy third trimester syncytium. In contrast, primary placental cells will spontaneously differentiate, making the expansion of a homogenous cell population more challenging, in addition to being more difficult to source. Stem cell technology has recently emerged to provide a cell source that couples the proliferative capacity of cell lines and the differentiation capacity of primary cells. Okae et al. recently derived proliferative trophoblast stem cells from human blastocysts capable of efficiently differentiating into a fused syncytium on tissue culture plastic (Figure S1, Supporting Information).^[^ ^15^ ^]^ These types of stem cell‐derived trophoblasts have been used to engineer organoids which recapitulate native placental villous polarity and architecture.^[^ ^16^, ^17^ ^]^ Most recently, Karvas et al. used these organoids to model SARS‐CoV‐2 and Zika viral infections in the placenta, and showed that cellular infection rates strongly depended on virus type and trophoblast subtype.^[^ ^16^ ^]^ Despite the capacity of placental organoids to model placental development, they are incapable of modeling barrier function. Further, these cultures usually lack perfusable vasculature which is necessary when modeling such a highly vascularized organ. The combination of stem cells and engineered vascular perfusion could unlock the exciting possibility of modeling different stages of pregnancy. Most existing models solely focus on simulating placental barrier function in the third trimester of pregnancy, at which point molecular transport is highest. However, modeling placental transport during early‐pregnancy could aid in identifying a safety window for certain drugs, such as antidepressants, whose prescription is sometimes necessary during early (or all) stages of pregnancy. Adverse effects of antidepressants have been shown to be drug type‐ and exposure‐dependent^[^ ^18^, ^19^ ^]^ and therefore may be influenced by placental permeability at the time of exposure. Often, doctors must weigh these risks against the mother's mental health, and would therefore benefit from more predictive trimester‐specific placental barrier models.^[^ ^20^ ^]^ To model these different stages of growth, an understanding of placental morphology and function at early‐ and late‐stage pregnancy is critical.

**Figure 1:**
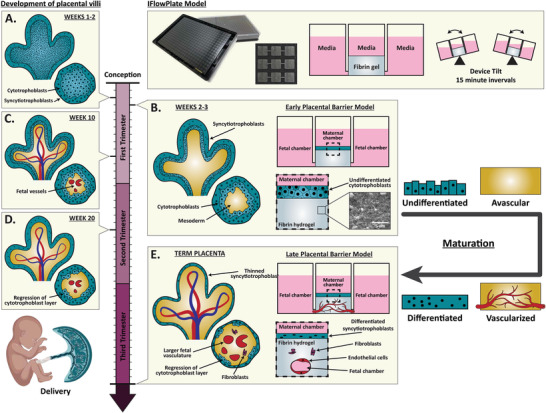
Timeline of development of placental villi and corresponding IFlowPlate models. A) During the first 2 weeks post‐implantation, primary chorionic villi consisting solely of cytotrophoblasts (CTs) lined with a layer of syncytiotrophoblasts (STs) sprout toward the maternal decidua. B) By the third week, the embryonic mesoderm will have grown into these branching structures to create what are known as secondary villi. This early placental stage of growth will be modeled using a monoculture IFlowPlate model consisting of a multilayered culture of cytotrophoblast‐like PSCs on a porous fibrin gel. C) Fetal vasculature gradually forms for the next few months until fetal circulation is established at week 10 of pregnancy. D) By week 20, the cytotrophoblast layer begins regressing, which increases transport of gases and nutrients toward the fetus to sustain increasing energy demands. E) As the pregnancy progresses, the syncytiotrophoblast layer thins and the branching, as well as vascularization of chorionic villi increase until delivery at around week 40. The term placenta is modeled using an IFlowPlate coculture model consisting of a differentiated syncytiotrophoblast monolayer cultured on a fibrin gel laden with fibroblast and endothelial cells which self‐assemble to form perfusable vasculature.

The early‐stage placental barrier is characterized by a lack of fetal circulation and by a thick trophoblastic barrier (**Figure** **1**A–C), whereas the later‐stage placenta consists of vascularized chorionic villi encased in a thin, fused trophoblast layer (syncytium) (Figure 1D,E), ideal for nutrient transport.^[^ ^21^ ^]^ In this study, we hypothesized that trophoblast differentiation and placental tissue vascularization can be modulated in vitro to model the early‐ and late‐stage placental barrier. To first model the early‐stage placental barrier, we cultured blastocyst‐derived placental stem cells (PSCs) on our previously published IFlowPlate384 platform, which contains 128 individual units made up of three wells interconnected by two microchannels (Figure 1B). The middle well of each unit contains a PSC‐derived cytotrophoblast barrier seeded on a fibrin gel matrix. To develop the late‐stage barrier model, perfusable vasculature was incorporated into the fibrin hydrogel and PSCs were differentiated into syncytiotrophoblasts on the gel surface (Figure 1E). We characterized gene expression, differentiation efficiency, and morphology of these cells pre‐ and post‐differentiation and compared them to the well‐established BeWo b30 trophoblast cell line. We showed that syncytiotrophoblast cells derived from our PSCs produced a more highly fused syncytium which more closely resembled the native syncytiotrophoblast. Next, we assessed how PSC differentiation altered barrier permeability and inflammatory cytokine secretion. We then assessed barrier permeability to dextran and insulin and showed how vasculature increased molecular clearance of small dextran molecules, which could explain efficient third‐trimester nutrient transport in vivo. Finally, we showed that the late‐stage model had reduced permeability to insulin when compared to its avascular equivalent, suggesting that the endothelial barrier plays an important role in selecting what molecules cross over to the fetus. In summary, our study demonstrated the importance of both differentiation efficiency and vascularization in designing physiologically representative in vivo placental barrier models. To our knowledge, this is the first in vitro platform that has coupled a highly differentiated syncytium with perfusable vasculature to model placental barrier permeability at different stages of pregnancy. Further, this is the first time it has been shown that the fetal endothelial barrier is the determining factor against insulin transport from the mother to the fetus.

## 2 Results

### 2.1 PSCs Exhibited Higher Rates of Fusion and Higher Secretion of hCG than BeWo b30 Model Cell Line

**Figure 2:**
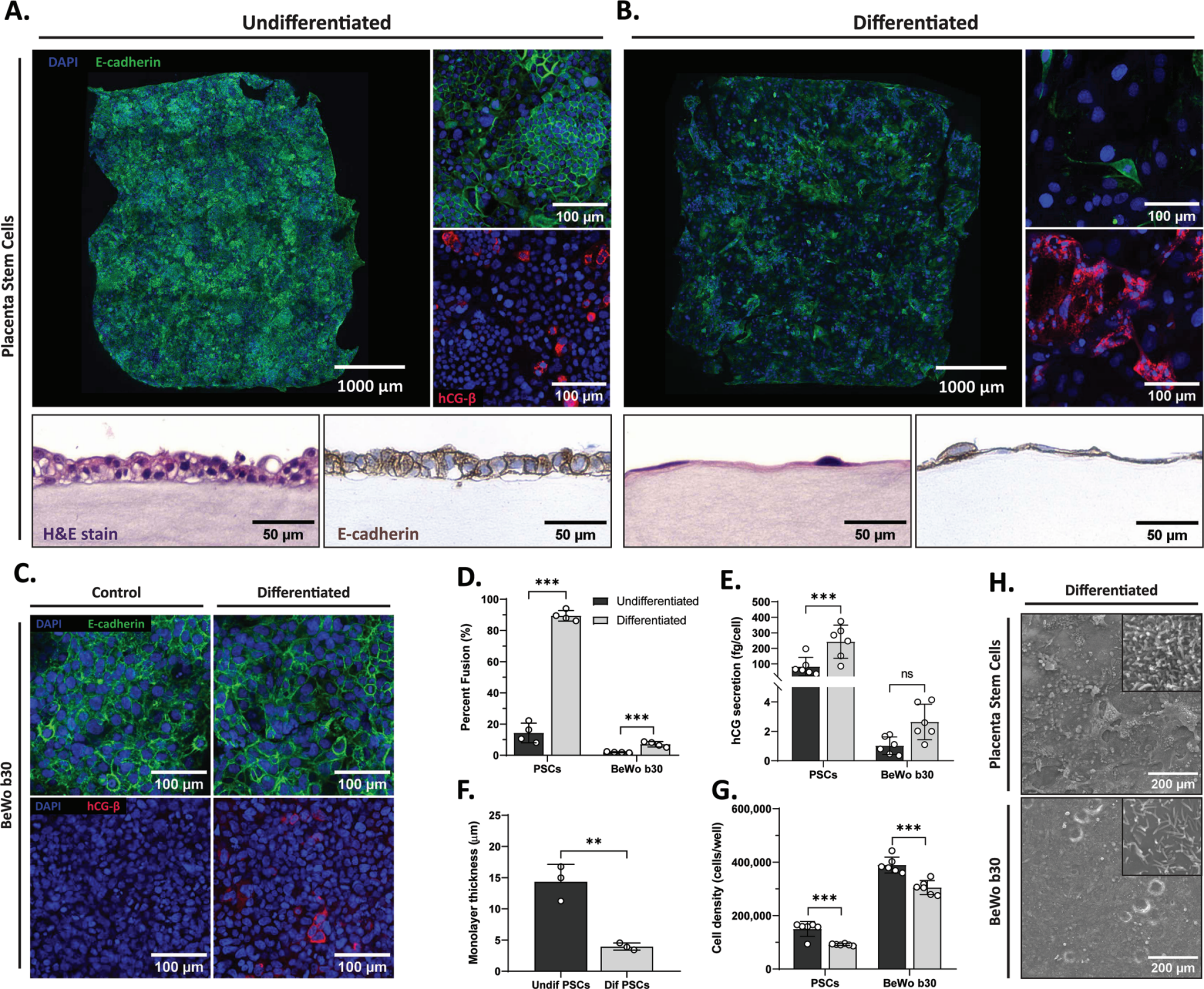
Characterization of PSC and BeWo b30 cell differentiation on fibrin gels indicates higher rates of fusion in PSCs. A) Undifferentiated PSCs were cultured on fibrin hydrogels for 8 days and stained for nucleic acids (DAPI, blue), cell adhesion marker e‐cadherin (green), and hCG‐β (red). Histology cross‐sections of entire gels were hematoxylin and eosin (H&E) stained and IHC stained, once again, for e‐cadherin (brown). B) PSCs were differentiated on fibrin hydrogels for 8 days and stained for nucleic acids (DAPI, blue), e‐cadherin (green), and hCG‐β (red). During differentiation, PSCs lost their localized e‐cadherin staining between cells, confirming successful trophoblast fusion. Histology cross‐sections of entire gels were stained for H&E and e‐cadherin (brown) and showed monolayer cell thinning as a result of differentiation. C) BeWo b30 cells were differentiated on fibrin hydrogels for 48 h and stained for nucleic acids (DAPI, blue), e‐cadherin (green), and hCG‐β (red). D) Percent fusion of undifferentiated and differentiated PSC and BeWo b30 cells (one‐way ANOVA, *N* = 4, ***p < 0.001). E) Secretion of hCG from undifferentiated and differentiated PSC and BeWo b30 cells over a span of 24 h (one‐way ANOVA, *N* = 3, ***p < 0.001). F) PSC monolayer thickness quantified from H&E‐stained histology cross‐sections (one‐way ANOVA, *N* = 3, **p < 0.01). G) Cell density of PSC and BeWo b30 cells cultured on fibrin gel in standard 384‐well plates (cells well^−1^) as measured via DNA quantification assay (one‐way ANOVA, *N* = 6, ***p < 0.001). H) Scanning electron microscopy images of microvilli on the apical surface of PSCs and BeWo cells differentiated on fibrin gel. PSCs appear to produce denser patches of microvilli when compared to BeWo b30 cells.

Blastocyst‐derived PSCs were compared against the well‐established trophoblast cell line, BeWo b30, to determine if they produced higher fusion rates necessary for late‐stage placental modeling. Both cell types were differentiated according to previously established protocols.^[^ ^15^, ^22^ ^]^ Briefly, PSCs were immediately seeded in differentiation medium and cultured for 8 days before analysis. BeWo b30 cells were first expanded for 2 days and then treated with 50 × 10^−6^ m forskolin and 50 ng mL^−1^ of epidermal growth factor (EGF) for 2 days of differentiation^[^ ^22^ ^]^ (**Figure** **2**). Longer‐term BeWo cell differentiation was attempted with the goal of increasing fusion rates, however monolayer integrity began to break down after more than 4 days of forskolin and EGF treatment, which may explain why they are normally only differentiated for up to 2 days. Fused cells were quantified via e‐cadherin staining where fusion was confirmed when at least three nuclei were present within a single e‐cadherin boundary. Fusion rates for differentiated PSCs (89.4 ± 3.4%) were over tenfold higher than the differentiated BeWo condition (7.1 ± 1.7%) (Figure 2A–D). In addition, the undifferentiated PSC control exhibited spontaneous fusion (14.3 ± .3%), whereas almost no spontaneous fusion was observed in the BeWo undifferentiated condition (1.9 ± 0.3%). Human chorionic gonadotropin (hCG) production correlated with fusion data, as expected, given that hCG is produced by fused villous syncytiotrophoblasts in vivo to maintain pregnancy (Figure 2E). The hCG concentrations measured in the PSC supernatant averaged at 125.2 ± 53.4 ng mL^−1^ for undifferentiated PSCs and 248.6 ± 112.6 ng mL^−1^ for differentiated PSCs. When compared to hCG level in the maternal serum during pregnancy, these values fall within the range of reported hCG concentrations^[^ ^23^ ^]^ between weeks 4 and 8 of pregnancy, as well as between weeks 18 and 40, when hCG levels begin to drop before parturition. However, because these concentrations are highly dependent on cell number and media volume, it is difficult to compare our measurement to in vivo. Further, terminal differentiation often results in proliferation arrest and thus, lower final cell numbers, which may skew results between conditions. We addressed this by normalizing hCG secretion to cell number. hCG secretion was highest in PSCs and increased with differentiation from 83.4 ± 59.1 to 242.7 ± 106.6 fg cell^−1^, whereas hCG secretion increased from 1.03 ± 0.60 to 2.64 ± 1.21 fg cell^−1^ in BeWo b30 cells. PSCs densities were 150 094 ± 27 960 cells well^−1^ for their undifferentiated condition and 91 657 ± 3447 cells well^−1^ when differentiated. BeWo cultures exhibited higher cell densities (389 347± 29 766 cells well^−1^ for undifferentiated and 305 066 ± 26 318 cells well^−1^ for differentiated) given their lack of contact inhibition^[^ ^24^ ^]^ and were able to stack into a multilayered cell barrier. Scanning electron microscope (SEM) images of differentiated PSCs and BeWo cells confirmed cell polarization by the presence of microvilli on the apical cell surface (Figure 2H). By visual inspection, PSCs appear to have higher microvillar densities than BeWo cells. Low microvillar densities have been observed in preeclamptic placental explants and it has been suggested that they may be an indicator of impaired trophoblast maturation.^[^ ^25^ ^]^ To further investigate the morphological transition between cytotrophoblast and syncytiotrophoblast cells, histology cross‐sections of PSCs were obtained (Figure 2A,B), and monolayer thickness was assessed (Figure 2F). The undifferentiated PSC control exhibited a multilayered structure, which is similar to what is seen in the early stages of pregnancy, whereas the differentiated tissue appears to be a multinucleated sheet covering the fibrin gel in a thin layer. In vivo, it has been shown that the trophoblast layer thins, from a 20–30 µm barrier in the first trimester to 2–4 µm in the third,^[^ ^26^ ^]^ to maximize feto‐maternal nutrient exchange. Similarly, our differentiated PSCs produced statistically thinner monolayers (3.95 ± 0.57 µm) when compared to their undifferentiated counterparts (14.33 ± 2.81 µm).

### 2.2 PSCs Exhibited Greater Similarity in Gene Expression to Native Syncytiotrophoblasts than BeWo b30 Model Cell Line

**Figure 3:**
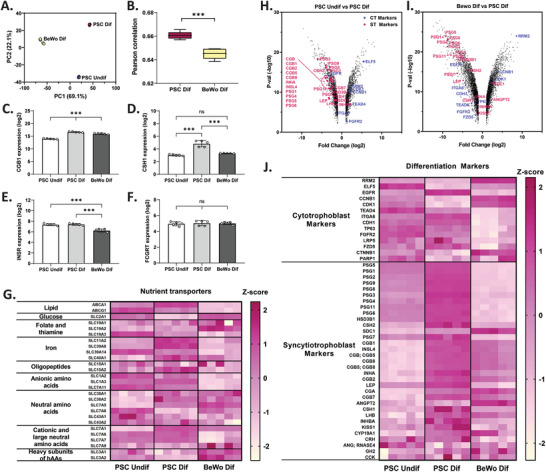
Gene expression analysis shows that PSCs more closely resemble primary syncytiotrophoblasts than BeWo b30 cells. A) Principal component analysis of gene expression of undifferentiated PSCs (PSC Undif), differentiated PSCs (PSC Dif), and differentiated BeWo b30 cells (BeWo Dif) (*N* = 5). B) Pearson correlation of PSC Dif and BeWo Dif cells with primary syncytiotrophoblast cultures^[^ ^27^ ^]^ (two‐tailed *T*‐test, *N* = 5, ***p < 0.001). C–F) Gene expression of hormone secretion genes C) hCG (CGB1) and D) hPL (CSH1), and important transporter genes for E) insulin (INSR) and F) immunoglobulin G (FCGRT) (one‐way ANOVA, *N* = 5, ***p < 0.001). G) Heat map displaying important placental nutrient transporter genes^[^ ^29^ ^]^ for all three experimental conditions. Gene expression levels were normalized by calculating *z*‐scores. Colors represent scaled expression values where magenta signifies high expression and white, low expression. H) Volcano plots comparing upregulated and downregulated genes from PSC Undif and PSC Dif cultures. Syncytiotrophoblast‐specific genes (ST markers) are highlighted in pink and cytotrophoblast‐related genes (CT markers) are highlighted in blue. Genes on the left of the zero axis are upregulated in the PSC Dif condition and those on the right of the zero axis are downregulated (*p* ≥ 0.05, fold change ≥ ±2). I) Volcano plots comparing upregulated and downregulated genes from BeWo Dif and PSC Dif cultures. Genes on the left of the zero axis are upregulated in the PSC Dif condition and those on the right of the zero axis are downregulated (*p* ≥ 0.05, fold change ≥ ±2). J) Heat map displaying CT and ST related markers for all three experimental conditions. Gene expression levels were normalized by calculating *z*‐scores. Colors represent scaled expression values where magenta signifies high expression and white, low expression.

Microarray sequencing was performed on both PSCs and BeWo b30 cells to determine if syncytiotrophoblast‐related genes were upregulated and cytotrophoblast genes were downregulated during differentiation. A principal component analysis was performed on the resulting gene expression data (**Figure** **3**A) and resulted in tight clusters of data points for each experimental condition, indicating low intersample variability. Next, gene expression data of PSC Dif and BeWo Dif conditions were compared to the gene expression of syncytiotrophoblast cells from third trimester placental explants^[^ ^27^ ^]^ by determining their Pearson correlation coefficient (Figure 3B). This coefficient represents the linear correlation between our experimental conditions and the primary explants, therefore the closer this variable is to 1, the more similar the datasets. The PSC Dif condition exhibited a Pearson correlation of 0.661 ± 0.003, which was statistically higher than that of the BeWo Dif condition (0.645 ± 0.004), suggesting that the gene makeup of differentiated PSCs is more similar to the primary tissue. Next, the expression of the genes responsible for the production of human chorionic gonadotrophin (hCG, CGB1) and human placental lactogen (hPL, CSH1) was shown to be highest in the PSC Dif condition (Figure 3C,D), as expected given that both hormones are mainly produced by the differentiated syncytium.^[^ ^23^, ^28^ ^]^ In contrast, both the insulin receptor gene (INSR) and neonatal Fc receptor gene (FCGRT), whose main function is to transport immunoglobulin G from maternal to fetal blood, remained unchanged in PSCs after differentiation (Figure 3E,F). Further, a list of crucial nutrient transporter genes^[^ ^29^ ^]^ was mapped (Figure 3G) and showed that lipid, iron, oligopeptide, cationic, and large neutral amino acid transporter genes were most highly expressed in the differentiated PSC condition. GLUT1 is the main glucose transporter across the human placenta^[^ ^30^ ^]^ and should increase with trophoblast fusion,^[^ ^29^ ^]^ however it was least expressed by the differentiated PSCs. This may be explained by the fact that hyperglycemia has been shown to downregulate GLUT1 transporters in human placental trophoblasts.^[^ ^31^, ^32^ ^]^ Cell culture media itself is hyperglycemic, and therefore cultures with higher cell densities, such as the undifferentiated PSCs (Figure 2G), and higher metabolisms deplete the glucose in the media quicker, thus lowering extracellular glucose levels and upregulating GLUT1 transporters. In contrast, choriocarcinoma cell lines have been shown to be unaffected by extracellular glucose concentrations,^[^ ^32^ ^]^ all of which would explain the difference in GLUT1 transporter gene SLC2A1. Similar heat maps were generated for thyroid transporters^[^ ^33^ ^]^ and pharmaceutically relevant drug transporters^[^ ^34^ ^]^ to demonstrate the extent at which fusion can impact trophoblast transport function (Figure S6, Supporting Information). The volcano plot of BeWo Dif versus PSC Dif had a larger spread (Figure 3H,I), which was expected given that the transcriptome was generated from two different cell types. Primary syncytiotrophoblast marker genes were more highly expressed in PSC Dif cells when compared to both PSC Undif and BeWo Dif, especially those coding for pregnancy hormones. In addition, primary cytotrophoblast markers were more highly expressed in the PSC Undif condition rather than the PSC Dif condition, suggesting that undifferentiated PSCs are more cytotrophoblast‐like. Interestingly, in addition to their higher ST marker expression, cells from the PSC Dif condition more highly expressed cytotrophoblast marker genes than BeWo Dif. However, this could be explained by the fact that a fraction of cells in the PSC Dif condition remains undifferentiated, and these cells could be more similar to primary cytotrophoblasts than undifferentiated BeWo cells, which are derived from a choriocarcinoma and would exhibit a more cancerous genome. The genes highlighted in the volcano plots were normalized as *z*‐scores to visualize their relative expression (Figure 3J). The PSC Dif condition exhibited highest normalized expression of most ST markers, most of which were hormone secretion genes^[^ ^35^ ^]^ (PSG family, CGB family, INSL4, INHA, LEP, CGA, ANGPT2, LHB, INHBA, KISS1, CSHL1, CRH, ANG, GH2, CCK). These findings are in agreement with the fact that the syncytium is the main site of polypeptide hormone secretion within the placenta.^[^ ^35^, ^36^ ^]^ In summary, the transcriptome profiling suggests that the PSC Undif condition is the most reminiscent of primary cytotrophoblasts and therefore ideal to include in our early‐stage barrier model, whereas the PSC Dif condition is most similar to the in vivo differentiated syncytium and thus ideal for late‐stage placental modeling.

### 2.3 Differentiation of the Early‐Pregnancy Placental Model Increased Monolayer Permeability to Dextran

**Figure 4:**
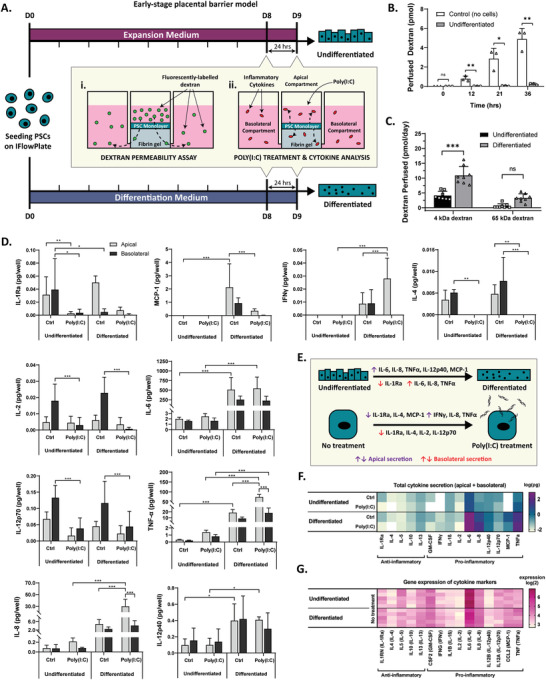
Differentiation of early‐stage placental barrier model triggers first trimester inflammatory response and change in permeability. A) Timeline for establishment of early‐stage placental barrier model (undifferentiated) and corresponding fused model (differentiated). i) Dextran permeability assay was performed by adding 65 kDa fluorescently labeled dextran into the central chamber and measuring its fluorescence in the adjacent chambers after 24 h. ii) To induce an inflammatory state in the early‐stage placental barrier model, poly(I:C) was added to the central chamber of the treated experimental groups. Cytokine secretion was analyzed in both the apical (maternal) and adjacent (fetal) chambers of differentiated and undifferentiated PSCs after 24 h. B) Timelapse of dextran perfusion into fetal chambers with and without cells. Dextran concentration continuously increases with time elapsed, whereas the PSCs were able to maintain resistance against 65 kDa permeation for at least 36 h without media change (two‐way ANOVA, *N* = 3, *p < 0.05, **p < 0.01). C) 65 and 4 kDa dextran permeability of undifferentiated and differentiated early‐stage barrier model. Differentiation increased PSC barrier permeability to 4 kDa dextran but did not affect permeability to 65 kDa dextran (two‐way ANOVA, *N* = 8, *p < 0.05, **p < 0.01). D) Cytokine secretion in apical (maternal) and basolateral (fetal) compartments of undifferentiated and differentiated PSCs with and without poly(I:C) treatment (two‐way ANOVA, *N* = 6, *p < 0.05, **p < 0.01, ***p < 0.001). E) Summary of the effects of PSC differentiation and poly(I:C) treatment on inflammatory cytokine secretion. F) Heat map of the effects of differentiation and poly (I:C) treatment on total inflammatory cytokine secretion (log(pg)) of PSCs cultured in early‐stage placental model. G) Expression (log2) of pro‐inflammatory and anti‐inflammatory cytokine gene markers in PSCs cultured in standard 6‐well plates.

Having confirmed the superiority of PSC over BeWo b30 differentiation, these cells were seeded on the IFlowPlate384 device. The central well is first cast with a fibrin hydrogel and PSCs are seeded on its apical surface to create our early‐pregnancy placental model. The adjacent wells serve as media storage and allow for its perfusion through the gel once the device is placed on a programmable tilter. This layout allows for compartmentalization of the maternal (center) and fetal (adjacent) compartments, which are separated by the fibrin gel representing the embryonic mesoderm (Figure 1B). PSCs were cultured on the device for 8 days before performing a dextran permeability assay (**Figure** **4**A). The hydrogel matrix alone (without cells) was highly permeable to 65 kDa dextran, allowing 2.86 ± 1.07 pmol to permeate into the adjacent wells after a 21 h incubation, and 4.90 ± 1.10 pmol, after 36 h (Figure 4B). In contrast, the PSC monolayer maintained near‐zero permeability at all timepoints between 0 and 36 h. To evaluate the effects of syncytial differentiation on monolayer permeability, a similar assay was performed on PSCs that had been differentiated on the device for 8 days (Figure 4A). Both TRITC‐labeled 65 kDa dextran and FITC‐labeled 4 kDa dextran were evaluated. PSC differentiation resulted in a size‐dependent increase in dextran permeability, where the 65 kDa dextran significantly increased from 0.62 ± 0.73 to 3.31 ± 1.40 pmol day^−1^ and 4 kDa dextran, from 4.13 ± 1.41 to 10.92 ± 3.00 pmol day^−1^ (Figure 4C).

### 2.4 PSC Differentiation Affected Inflammatory Cytokine Secretion

Pregnancy triggers an inflammatory state in the human body.^[^ ^37^ ^]^ As pregnancy progresses, blood concentration of many pro‐ and anti‐inflammatory cytokines will fluctuate in a tightly coordinated immune response to maintain and support the pregnancy. Pro‐inflammatory (GM‐CSF,^[^ ^38^ ^]^ IFNγ,^[^ ^39^ ^]^ IL‐1β,^[^ ^40^ ^]^ IL‐2,^[^ ^41^ ^]^ IL‐6,^[^ ^42^, ^43^ ^]^ IL‐8,^[^ ^44^ ^]^ IL‐12p40,^[^ ^43^ ^]^ IL‐12p70, MCP‐1,^[^ ^45^ ^]^ TNF‐α^[^ ^43^ ^]^) and anti‐inflammatory (IL‐1Ra,^[^ ^46^ ^]^ IL‐4,^[^ ^47^ ^]^ IL‐5,^[^ ^48^ ^]^ IL‐10,^[^ ^44^, ^47^ ^]^ IL‐13^[^ ^48^ ^]^) cytokines from both undifferentiated and differentiated barrier models were quantified to determine if we could simulate these cytokine profiles (Figure 4A). In addition, both undifferentiated and differentiated tissues were treated with viral mimic, poly(I:C), for 24 h, as a positive inflammatory control. Differentiation triggered an increase of pro‐inflammatory cytokines (IFNγ, IL‐6, IL‐8, IL‐12p40, MCP‐1, TNF‐α) and a decrease in anti‐inflammatory cytokine IL‐1Ra (Figure 4D–F). Further, treatment with 10 µg mL^−1^ of poly (I:C) decreased the secretion of MCP‐1, IL‐2, IL‐4, IL‐1Ra, IL‐12p70 and increased the secretion of pro‐inflammatory cytokines, IL‐8, TNF‐α, IFNγ, indicating our model can provide a robust inflammatory response to external stimuli. Interestingly, poly(I:C) treatment did not affect IL‐6 secretion rates, despite being present at high levels in the supernatant. This could potentially be due to the fact that IL‐6 secretion is modulated by both matrix stiffness^[^ ^49^, ^50^ ^]^ and fibrin matrix degradation^[^ ^51^ ^]^ in some cell types, which may have dampened any poly(I:C)‐mediated increase in IL‐6. In contrast, PSC differentiation increased IL‐6 secretion by over 100‐fold in basolateral and apical compartments. This was consistent with findings that syncytiotrophoblasts more highly stain for IL‐6^[^ ^52^ ^]^ and with longitudinal studies showing that IL‐6 levels in maternal sera increase with gestational age.^[^ ^53^ ^]^ Along with IL‐6, TNF‐α was one of the most highly affected pro‐inflammatory cytokines in this study. Undifferentiated and untreated PSCs secreted near‐zero amounts of TNF‐α into both compartments (0.32 ± 0.08 pg for apical and 0.23 ± 0.05 pg for basolateral) and the differentiated, poly(I:C)‐treated cells secreted the most TNF‐α intro the apical chamber (74.4 ± 13.5 pg). This is expected, given that TNF‐α secretion continuously increases throughout pregnancy in normal weight women.^[^ ^53^, ^54^, ^55^ ^]^ TNF‐α has been shown to induce IL‐8 expression in multiple cell types^[^ ^56^, ^57^ ^]^ which explains how trophoblast IL‐8 secretion followed an identical trend. IL‐8 increased most substantially with differentiation, however poly(I:C) treatment appeared to trigger an increase in the apical compartment exclusively. This increase was most notable in differentiated cells, where IL‐8 secretions increased from 4.83 ± 2.15 to 29.9 ± 12.0 pg.

In vivo, MCP‐1 is produced in large quantities by trophoblast cells.^[^ ^58^, ^59^ ^]^ It has been shown that MCP‐1 secretion into the maternal compartment of perfused placental explants is much higher than its secretion into the fetal compartment.^[^ ^59^ ^]^ This suggests that the bulk of MCP‐1 secretion may occur within the syncytiotrophoblast cells, which are in direct contact with the maternal circulation. This is reflected in our model where MCP‐1 secretion increased with PSC differentiation (from undetectable levels to 2.13 ± 1.77 pg for apical and 0.94 ± 0.40 pg for basolateral). Interestingly, MCP‐1 then decreased with poly(I:C) treatment (0.36 ± 0.16 pg for apical and 0.02 ± 0.02 pg for basolateral). MCP‐1 is both responsible for maintaining M1/M2 balance of macrophages and regulating trophoblast invasion of the placenta.^[^ ^58^ ^]^ This reduction in MCP‐1 secretion by the differentiated syncytiotrophoblasts could be signaling to extravillous trophoblast cells to halt invasion into the “infected” maternal tissue. IFNγ levels rose with differentiation from below the detectable limit to 0.01 ± 0.01 pg apically and 0.01 ± 0.01 pg basolaterally. When treated with poly(I:C), apical secretion further increased to 0.03 ± 0.02 pg, whereas basal secretion dropped back under the detectable limit. During early pregnancy, IFNγ is mostly produced by the uterus to initiate the endometrial vascular remodeling necessary for the growth of the fetus,^[^ ^60^ ^]^ however it has also been shown to be produced by human trophoblast cells.^[^ ^61^ ^]^ This early transient production of IFNγ is believed to be necessary in preventing maternal immune activation against the semiallogeneic fetus.^[^ ^62^ ^]^ In addition, IFNγ inhibits invasion of extravillous trophoblasts in the first trimester, which is believed to be necessary in preventing excessive invasion during implantation, leading to the dangerous condition of placenta accreta.^[^ ^63^, ^64^, ^65^ ^]^ Anti‐inflammatory IL‐4 secretion averages ranged between 0.003 and 0.008 pg for untreated conditions and were not significantly affected by differentiation or polarity. This finding was surprising given that, along with IL‐6 and IL‐7, IL‐4 has been shown to stimulate hCG release within the placenta.^[^ ^66^, ^67^ ^]^ IL‐4 then decreased to below detectable limits with poly(I:C) treatment, as expected, given its anti‐inflammatory properties. Basolateral levels of IL‐12p40 remained constant throughout all four conditions, whereas apical levels of IL‐12p40 increased with PSC differentiation in both poly(I:C) treated (0.099 ± 0.083 to 0.409 ± 0.036 pg) and nontreated conditions (0.096 ± 0.078 to 0.399 ± 0.205 pg).

IL‐2 is primarily known as a T cell growth factor and is responsible for the development of peripheral immune cells.^[^ ^68^, ^69^ ^]^ Although levels of IL‐2 secretion were low and did not change with differentiation, apical‐basal polarity was observed. Basolateral secretion (0.018 ± 0.010 and 0.023 ± 0.010 pg) was 3.6‐fold and 3.8‐fold higher than the apical secretion (0.005 ± 0.003 and 0.006 ± 0.003 pg) in the undifferentiated and differentiated untreated groups, respectively. This polarity was also observed with poly(I:C) treatment, where changes in IL‐2 levels were only significant in the basolateral compartment while apical IL‐2 levels remained constant. Despite IL‐2 conventionally being categorized as a pro‐inflammatory cytokine, it has been shown that IL‐2 reduces inflammation during pregnancy and can normalize certain symptoms in rats suffering from placental ischemia.^[^ ^70^, ^71^ ^]^ If IL‐2 acts as an anti‐inflammatory cytokine within the placenta, this may explain why its basolateral secretion was reduced with poly(I:C) treatment. Similarly, basolateral IL‐12p70 levels dropped with poly(I:C) treatment in both undifferentiated (0.133 ± 0.038 to 0.038 ± 0.032 pg) and differentiated conditions (0.116 ± 0.067 to 0.044 ± 0.047 pg). IL‐12 is not only pro‐inflammatory, but antiangiogenic and therefore may be important in regulating fetal vascular formation throughout villous development,^[^ ^72^ ^]^ which would explain the higher baseline IL‐12p70 secretion values in the basolateral fetal compartment. Finally, there were no significant changes in apical or basolateral secretion of GM‐CSF, IL‐1β, IL‐4, IL‐5, IL‐10, and IL‐13 as a result of differentiation or poly(I:C) treatment (Figure S7, Supporting Information). Overall, cytokine secretion was more strongly affected by PSC differentiation than to poly(I:C) treatment. This may be due to differences in treatment times (48 h for poly(I:C) vs 8 days for differentiation) or mass transport differences between media supplements (poly(I:C) vs forskolin) when crossing the placental barrier. However, we believe that the high level of inflammation caused by the fusion of PSCs is reminiscent of the first trimester of pregnancy, during which cytotrophoblast cells are rapidly proliferating and fusing to form the syncytium. At this early stage, trophoblasts will secrete chemokines which will recruit and reprogram the mother's immune system to shield the fetus from both the maternal immune system itself and exogenous threats.^[^ ^73^, ^74^ ^]^ Because the baseline inflammation during pregnancy is already high, health risks to the mother and fetus are increased. Viral infections, smoking, excessive weight gain, and a host of other factors may increase inflammation and result in adverse pregnancy outcomes, driving the need for informative in vitro models such as this one.

### 2.5 Cytokine Gene Expression Showed Similar Trends to Cytokine Secretion Data

Many trends observed in the cytokine secretion data were confirmed using gene expression data of PSCs. Microarray sequencing was performed on undifferentiated and differentiated PSCs, and gene marker expression of the 15 inflammatory cytokines studied in the cytokine secretion panel was more closely examined (Figure 4G). Expectedly, gene marker expression for pro‐inflammatory cytokines IL‐8, IL‐12p40, and TNF‐α increased significantly with PSC differentiation by factors of 1.09, 1.09, and 1.17, respectively, which agreed with their corresponding secretion data. IL1RN (IL‐1Ra) expression increased modestly with differentiation by 1.10‐fold. Although a similar increase in IL‐1Ra secretion was observed in the apical compartment of our differentiated IFlowPlate cultures, it did not achieve significance (*p* = 0.53). Similarly, IL10 (IL‐10) expression increased slightly with differentiation by a factor of 1.12, despite IL‐10 secretion remaining constant. The only discrepancy between the two datasets was for pro‐inflammatory cytokine IL‐6 that increased with differentiation, but the corresponding gene marker expression decreased by a factor of 1.16. In vivo, IL‐6 expression has been shown to be most highly expressed in cytotrophoblasts and will decrease as cell fusion occurs,^[^ ^75^, ^76^ ^]^ similar to what was observed in our microarray expression data. In contrast, it has also been shown that, when compared to cytotrophoblasts, syncytiotrophoblasts more strongly stain for IL‐6,^[^ ^52^ ^]^ which is consistent with our cytokine secretion data. This discrepancy can be explained by the fact that mRNA often does not fully represent protein secretion, especially when dealing with transient systems.^[^ ^77^, ^78^, ^79^ ^]^ Alternatively, this difference could be explained by the differences in culture substrates used between experiments (gene expression on a 6‐well plate and cytokine secretion on IFlowPlate), which has been shown to affect inflammatory cytokine secretion.^[^ ^49^, ^50^ ^]^ Further, there were no significant differences in cytokine gene expression of anti‐inflammatory cytokines IL‐4, IL‐5, and IL‐13, as well as pro‐inflammatory cytokines GM‐CSF, IFNγ, IL‐1β, IL‐2, IL‐12p70, and MCP‐1, which agrees with the cytokine secretion findings. The significant increase in MCP‐1 secretion triggered by PSC differentiation was not reflected in the gene expression data (*p* = 0.23), however it appears that CCL2 (MCP‐1) gene expression trended in that direction. Overall, gene expression of the 15 inflammatory cytokines examined largely agreed with the results of the cytokine secretion panel and showed that PSC differentiation directs inflammatory cytokine release.

### 2.6 Late‐Pregnancy Barrier Model Achieved High PSC Differentiation Rates

**Figure 5:**
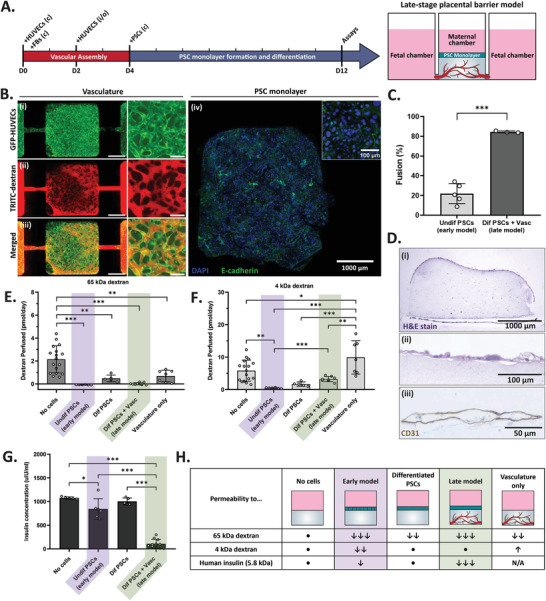
Late‐stage placental barrier model exhibits altered permeability compared to early‐stage model. A) Timeline for establishment of late‐stage placental barrier model. HUVECs and fibroblasts are cast into the central gel of the device and cultured for 2 days before adding HUVECs into fetal compartments to line connecting channels. PSCs are then seeded into the maternal chamber at day 4 and immediately differentiated for 8 days before permeability assays are performed. B) Imaging of late‐stage placental barrier model. i) GFP‐labeled HUVECS (green) and fibroblasts self‐assembled into fetal vasculature and dextran (red) perfused across fetal channels at end of culture (D13) (100 µm scale bar). ii) Differentiated PSC monolayer from late‐stage model stained for nucleic acids (DAPI, blue) and for e‐cadherin (green) to demonstrate high degree of trophoblast fusion. C) H&E‐stained histological cross‐sections of i) whole fibrin gel with apical PSC monolayer and perfusable vasculature at base. Higher magnification images of ii) H&E‐stained fused PSC monolayer and iii) CD31‐stained HUVECs (brown) forming vascular structures. D) Permeability of 65 kDa dextran across different barrier models (two‐way ANOVA, *N* = 5 to *N* = 17, *p < 0.05, ***p < 0.001). E) Permeability of 4 kDa dextran across different barrier models (two‐way ANOVA, *N* = 5 to *N* = 17, *p < 0.05, ***p < 0.001). F) Insulin concentrations in supernatant from basolateral (fetal) compartments of late‐stage placental barrier model (two‐way ANOVA, *N* = 3 to *N* = 11, *p < 0.05, ***p < 0.001). Apical insulin concentrations were statistically similar between all culture conditions, whereas vascularization of model caused a reduction in insulin transport into the basolateral compartment. G) Table summarizing transport findings across all models relative to the No cell control (•).

Vascularization is one of the defining characteristics of the placental organ and evolves throughout pregnancy. When chorionic villi initially form, fetal vasculature is underdeveloped and does not yet allow fetal blood circulation, which is vital to the efficient transport of nutrients to the developing fetus. This may explain why feto‐maternal nutrient transport only commences at the onset of fetal circulation at around week 12 of gestation.^[^ ^80^ ^]^ Therefore, perfusable vasculature is a defining feature that distinguishes between early‐ and late‐stage placental transport. Having established a model for early‐stage pregnancy with undifferentiated cytotrophoblasts, next we incorporated vasculature and differentiated syncytiotrophoblasts to model late‐stage pregnancy. To do this, human endothelial and fibroblast cells were incorporated into the fibrin hydrogel before being cast into the IFlowPlate (Figure 1E). Cells were cultured in the platform for 4 days to allow for vascular self‐assembly, then PSCs were seeded onto the apical surface of the gel, similar to the early‐pregnancy model (**Figure** **5**A). Once PSCs were added, the IFlowPlate was switched to compartmentalized media culture in which PSC media was maintained in the central well and endothelial cell media, in the adjacent inlet and outlet wells. A culture media tolerance assay confirmed that both cell types were able to grow in mixed media (Figure S8, Supporting Information). Cultures were maintained with daily media changes for 8 more days to allow for adequate PSC fusion. Vascular perfusion was confirmed immediately before or after permeability measurements were taken by introducing fluorescently labelled dextran into the inlet compartment and monitoring its perfusion throughout the vasculature and into the outlet channel (Figure 5B). Tissues were fixed and stained for 4′,6‐diamidino‐2‐phenylindole (DAPI) and e‐cadherin to assess PSC fusion (Figure 5B). Fusion rates of PSCs were very similar in the late‐stage model (Dif PSCs + Vasc, 84.4 ± 1.0%, Figure 5C) than in differentiated monoculture (89.4 ± 3.4%, Figure 2D) which suggested that compartmentalized media and endothelial cell coculture did not heavily impact syncytiotrophoblast fusion rates. As expected, PSC fusion rates in our late‐stage model were significantly higher than those of our early‐stage barrier model (Undif PSCs, 21.9 ± 10.1%, Figure 5C), which still exhibited some spontaneous fusion. Despite this modest reduction, fusion rates were still far improved from those of BeWo b30 cells in ideal culture conditions (7.1 ± 1.7%, Figure 2D). Histology cross‐sections of the late‐stage tissue show that the differentiated PSCs formed of a single, thin cell monolayer. Monolayer thickness (Figure 5D) was comparable to that of differentiated PSCs on fibrin (Figure 2B), once again confirming successful PSC differentiation into a fused syncytium. Endothelial cells were immunohistochemistry (IHC) stained for CD31, an endothelial marker for vascular differentiation, which highlighted the luminal structure of the self‐assembled vasculature.

### 2.7 Vascularization Altered Dextran Permeability of Barrier Model in a Size‐Dependent Manner

After a total of 12 days in culture, barrier permeability was assessed in both vascularized and nonvascularized models via dextran and insulin permeability assays (Figure 5A). Barrier measurements were always performed on the same day (D12) to control for any differences in vascular morphology and their resulting effects on molecular transport. Similar to vascularized tissues, nonvascularized cultures were maintained in compartmentalized media to control for culture media effects and isolate the effects of vascularization on barrier permeability. Vascularization affected dextran permeability in a size‐dependent manner. Vasculature significantly decreased 65 kDa dextran permeability (Figure 5E) from 2.15 ± 1.18 (no cell) to 0.69 ± 0.52 pmol day^−1^ (Vasc only) through the hydrogel matrix. Further, lower permeability results were also observed with the addition of vasculature to the differentiated PSC model (0.47 ± 0.29 for Dif PSC condition to 0.01 ± 0.10 pmol day^−1^ for Dif PSC + Vasc condition), however they did not prove to be significant (*p* = 0.91). In contrast, vascularization significantly increased 4 kDa dextran diffusion (Figure 5F) into the adjacent wells when compared to the “no cell” control group (5.83 ± 3.25 for no cell condition to 9.91 ± 5.16 pmol day^−1^ for vasculature only condition, *p* = 0.04) and appeared to result in higher permeability values for the differentiated PSC condition (1.70 ± 0.67 for Dif PSC condition to 3.23 ± 0.80 pmol day^−1^for Dif PSC + Vasc condition). These results suggest that the addition of vasculature acts as an extra barrier against 65 kDa dextran permeability, whereas it allows for faster clearance of smaller 4 kDa dextran molecules, thus increasing its transport rates into the adjacent compartments. These findings highlight the importance of incorporating vasculature in placental models for fetal toxicity studies, given that small‐molecule drugs will more rapidly permeate vascularized tissue. Therefore, nonvascularized placental barrier models may falsely deem a drug safe for use during pregnancy. This result also aligns with the functional role of vasculature in the placenta, which is to accelerate mass transport as the placenta becomes more highly vascularized in late‐stage pregnancy. In previous placenta‐on‐a‐chip studies, the endothelium functions as an additional barrier and hinders mass transport, without capturing the clearance effects of perfusable vasculature. Here, we clearly showed that a matrix that is more extensively vascularized will improve mass transport, at least for small molecules.

### 2.8 Vascularization Decreased Barrier Permeability to Insulin

Ex vivo studies have shown that the placenta is impermeable to insulin at physiological levels, which allows diabetic patients to continue their insulin treatments throughout pregnancy. Here, we tested our barrier models to determine whether PSC differentiation and vasculature have a significant effect on insulin transport from the maternal to fetal compartments (Figure 5G). Interestingly, we first found that our undifferentiated (early‐stage) condition only slightly decreased (843.8 ± 215.6 µIU mL^−1^) the permeation of insulin into the fetal compartments, whereas the differentiated PSC monolayer (1003 ± 73.7 µIU mL^−1^) did not present any type of resistance against insulin passage. The amount of insulin that crossed into the fetal chambers was statistically similar to our “no cell” control (1076 ± 23.9 µIU mL^−1^) which consisted of a fibrin gel alone. The undifferentiated PSC monolayer (early‐stage model) and the differentiated PSC monolayer had statistically similar insulin permeation which agreed with our earlier findings that the INSR expression does not change with PSC differentiation. In contrast, the addition of vasculature in our late‐stage placental model drastically reduced insulin permeation (108.1 ± 95.7 µIU mL^−1^) similar to in vivo. It is often assumed that the trophoblast barrier is the most selective when it comes to feto‐maternal transport, however in ex vivo placental perfusion studies, researchers are not able to distinguish which cell layer provides resistance against molecular passage. Similar to trophoblast cells, human umbilical vein endothelial cells (HUVECs) and fetal endothelial cells are also known to express INSR,^[^ ^81^, ^82^, ^83^ ^]^ however fetal endothelial cells have been shown to express the gene at much lower rates than first trimester trophoblast cells,^[^ ^83^ ^]^ which may explain why the endothelial cells provided more of a barrier against insulin transport from the maternal to fetal compartment of our late‐stage model. These results suggest that the presence of an endothelial layer is critical to insulin transport modeling and may significantly affect the safety ratings of molecules tested in in vitro type placental barrier models (Figure 5H).

## 3 Discussion

The placenta is a rapidly evolving, transient organ which is considered the main site of nutrient and gas exchange between the mother and the fetus. In this work, we engineered a hydrogel‐based culture model which was capable of simulating early‐ and late‐stage placental function with the careful modulation of differentiation and vascularization. We first characterized the differentiation capacity of our blastocyst‐derived trophoblast stem cells on a fibrin hydrogel by quantifying trophoblast fusion efficiency, hCG secretion, and monolayer thickness. During the early weeks of pregnancy, the corpus luteum secretes pregnancy‐maintaining hormones until placental maturation occurs. Trophoblast cells often only have the capacity to produce hormones such as progesterone and placental growth hormone in the more mature placenta,^[^ ^84^ ^]^ with the exception of hCG. Low levels of hCG are detectable in maternal blood and urine as early as 2 weeks after ovulation. hCG continues to almost double every few days until it reaches its peak concentration at the end of the first trimester.^[^ ^85^ ^]^ The undifferentiated PSCs exhibited an 80‐fold higher baseline hCG production compared to the BeWo b30 cell line and further increased their secretion with differentiation, more closely adhering to this physiological process. Microarray gene sequencing was then performed on both PSCs and BeWo cells, which confirmed a higher expression of fusion‐related genes in PSCs.

Similar to hormone production, the placenta takes over pregnancy nutrient exchange from the yolk sac at the end of the first trimester.^[^ ^86^ ^]^ The early‐stage placental villi therefore feature a structure less complex than the term placenta and are characterized by an embryonic mesoderm sheathed in multiple layers of cytotrophoblasts and a single syncytiotrophoblast layer. We constructed an early‐stage placental barrier model consisting of an undifferentiated PSC monolayer cultured onto the fibrin gel of our lab's IFlowPlate device. Most importantly, the permeability of the placenta is continuously evolving throughout the pregnancy. Barrier permeability was assessed in this model using fluorescently labeled dextran and compared to the differentiated equivalent, which exhibited size‐dependent permeability differences. We showed that cytotrophoblast fusion slightly increases monolayer permeability to small molecules, and therefore we believe that the syncytiotrophoblast layer present in early‐stage villi would offer negligible resistance to molecular diffusion compared to the multiple layers of cytotrophoblast cells. This assumption is supported by a study that found that the cytotrophoblast layer of the rabbit placenta offers the most amount of diffusional resistance against lipid‐insoluble molecules compared to the syncytiotrophoblast.^[^ ^87^ ^]^ To do so, Faber and Stearns used the tracer‐dilution method, which consists of perfusing both permeable and impermeable tracers across the maternal and fetal compartments and measuring the recovery over time. More recently, it has been shown that the syncytiotrophoblast layer contains trans‐syncytial nanopores which increase molecular diffusion across this barrier.^[^ ^88^ ^]^ As a result, the multilayered undifferentiated PSC culture should be a valid model to represent early pregnancy.

Cytotrophoblasts perform the bulk of their differentiation into syncytiotrophoblasts in the first trimester and continue this process throughout the entire pregnancy to replenish the syncytium. The trophoblast differentiation process triggers the release of pregnancy hormones such as hCG, however it also coincides with an increase in inflammatory cytokines within the maternal sera.^[^ ^89^ ^]^ We showed that PSC fusion increased the secretion of many pro‐inflammatory cytokines and decreased the secretion rates of anti‐inflammatory IL‐1Ra. Interestingly, this increase is much larger than that resulting from poly(I:C) treatment, indicating a considerable inflammatory state. This increase in inflammation is observed in the first trimester of healthy pregnancies, where the maternal immune system is working synergistically with the trophoblast cells, which are secreting inflammatory cytokines, to facilitate implantation and fetal growth. When dysfunction occurs in immune signaling between the fetus and mother, risk of miscarriage increases,^[^ ^90^ ^]^ which is one of the reasons why the first trimester has the highest risk of miscarriages.^[^ ^91^ ^]^ The inflammation observed in early pregnancy decreases once in the second trimester. By transitioning the differentiated cultures back to expansion medium, it may be possible to maintain syncytial fusion while reducing inflammatory cytokine production and thus simulating this first‐to‐second trimester transition. Interestingly, most of the factors in the differentiation media were already present in the expansion media, however at lower amounts, except for forskolin, which has been shown to have anti‐inflammatory properties^[^ ^92^, ^93^ ^]^ and knockout serum replacement (KSR) which should not have any inflammatory effects as it is a nutrient supplement. This suggests that the combination of these factors, when inducing cell fusion, triggers an inflammatory state in the PSCs which may be necessary in vivo, given the inflammatory state of the body in the first trimester.

The placenta cannot perform most of its barrier function in the first trimester, however its permeability is selective and increases with gestation time throughout the second and third trimesters.^[^ ^94^ ^]^ This is a result of several structural changes in the chorionic villi and morphological changes in the trophoblast cells that compose it. As the placenta matures, vascularization will increase, and fetal blood vessels will thicken to accommodate more blood perfusion. At the cellular scale, cytotrophoblasts regress and syncytiotrophoblasts thin toward the end of pregnancy, which reduces the barrier thickness between mother and fetus and further increases transport. In addition, expression of hormone receptors changes when cytotrophoblasts differentiated into syncytiotrophoblasts^[^ ^95^, ^96^ ^]^ which may have downstream effects on placental permeability.^[^ ^97^ ^]^ These phenomena work synergistically to modulate feto‐maternal placental transport. We designed a late‐stage placental barrier model by incorporating self‐assembled vasculature into the fibrin hydrogel of our IFlowPlate device and differentiating the PSC monolayer. Including vascular perfusion within our model altered its permeability and clearance of multisized dextran molecules, as well as insulin. The addition of perfusable vasculature appeared to decrease 65 kDa dextran transport and increase 4 kDa dextran transport from the maternal to fetal chambers. These findings suggest that the self‐assembled vessels provided a significant barrier against larger molecules, however the resulting vascular perfusion permitted the faster clearance of smaller molecules. This increased clearance rate was largely due to the increased luminal flow velocity within the vessels, ranging from 13.1 to 338.9 µm s^−1^, depending on the vessel dimensions,^[^ ^98^ ^]^ when compared to flow velocity through the gel alone (3.98 ± 2.22 µm s^−1^ (Figure S3F,G, Supporting Information).

Interestingly, insulin, which is of similar size (5808 Da) to the smaller dextran, permeated the barrier more slowly when vasculature was added to the system (Figure 5G). Human insulin and many of its analogs have been shown not to cross the human placental barrier at physiological levels.^[^ ^99^, ^100^, ^101^, ^102^, ^103^, ^104^ ^]^ Here, the PSC media in the maternal compartment (apical) contained supraphysiological levels of insulin from animal serum, which produced a larger concentration gradient across the placental barrier. Despite this, the vascularized late‐stage placental barrier model was capable of maintaining a low concentration of insulin in the fetal compartments (basolateral). Interestingly, the corresponding avascular model did not provide any significant resistance against insulin permeation into the fetal compartment. These findings indicate that fetal vasculature may function as the main barrier against insulin transfer to the fetus. However, it is also possible that the endothelial or fibroblast cells within the gel matrix are binding or degrading the insulin, or that fibroblast deposition is stiffening the matrix,^[^ ^105^, ^106^ ^]^ thus reducing molecular transport. Although we could not fully control for matrix property changes, we made certain to test the groups on the same day to eliminate culture length effects on matrix degradation. Most ex vivo experiments which have characterized insulin permeability use placental explants which cannot uncouple the effects of the trophoblast, stromal, and endothelial cell layers. Regardless of the mechanism of action at play, the incorporation of vasculature into the late‐stage barrier model produced insulin permeability data more reminiscent of the in vivo placental environment. This work highlights the importance of controlling both trophoblast differentiation and barrier vascularization when modeling the placental barrier.

As with all in vitro models, there are certain limitations to address. First, the flow direction of vascular perfusion in our model is bidirectional instead of the unidirectional flow of blood vessels, which may impact endothelial cell morphology and certain inflammatory pathways.^[^ ^107^ ^]^ However, this bidirectional flow pattern still permitted the self‐assembly of fibroblast and endothelial cells into functional vessels reminiscent of human vasculature. Further, this platform utilizes fibrin as its biopolymer hydrogel matrix instead of basement membrane proteins such as collagen or laminin, which are the main components of the placental extracellular matrix (ECM).^[^ ^108^ ^]^ Although collagen gels would have provided a more physiologically realistic ECM environment for trophoblast cells, they degrade and contract significantly over time.^[^ ^109^ ^]^ In contrast, fibrin degradation can easily be controlled with the addition of aprotinin to culture media^[^ ^110^ ^]^ and provide a stable porous substrate that supports cell attachment and endothelial cell growth. Further, they are conducive to collagen and laminin deposition by fibroblast cells, which alter the cellular microenvironment and promote 3D cellular organization.^[^ ^111^ ^]^ Next, the use of lung fibroblasts and HUVECs, whose self‐assembly has previously been optimized in our IFlowPlate platform,^[^ ^98^ ^]^ is not native to the human placenta and may result in vessels with slightly different barrier functions. Optimizing vessel self‐assembly in this platform using fetal endothelial cells and placental fibroblasts is the next step in elevating the physiological relevance of this model. Finally, fetal vasculature is in close proximity to the trophoblast barrier in native tissues, which is not reflected in our model. However, this distance may be optimized by reducing hydrogel thickness or by increasing vascular presence in the top half of the gel. To do this, the plate can be inverted or the prepolymer hydrogel viscosity can be tuned to prevent cell precipitation during gelation. In addition, the endothelial and fibroblast cell density within the fibrin gel can be varied to either increase or decrease the extent of vascularization, more finely tuning the developmental stage which this device is simulating.

The versatility of our IFlowPlate platform allows us to make further potential improvements to more closely mimic certain aspects of the native placental barrier. In this work, we triggered PSC fusion into syncytiotrophoblasts by culturing them in differentiation medium containing forskolin, a cAMP‐activator extracted from plant root, which is not present in the human placenta. In vivo, this cAMP pathway is the main route toward fusion, and it is activated by various chemical and mechanobiological factors, whose mechanisms are largely still unknown.^[^ ^112^, ^113^ ^]^ Fluid shear stress has been shown to increase levels of intracellular cAMP in primary human syncytiotrophoblast cells^[^ ^114^ ^]^ and to significantly increase fusion rates of rabbit trophoblast stem cells without the presence of forskolin.^[^ ^115^ ^]^ These findings suggest that incorporating fluid flow into the apical chamber of our device could trigger shear stress‐induced PSC fusion and bypass any nonphysiological side effects of forskolin treatment on our system. Moreover, substrate mechanical stiffness has been shown to affect fusion rates of BeWo cells^[^ ^116^ ^]^ and can easily be tuned in our model with the goal of triggering mechanically induced syncytialization. Finally, native placental villi form branched structures resulting in large surface areas which are conducive to nutrient transport. These structures may be incorporated into our late‐stage barrier system using an already established hydrogel patterning technique.^[^ ^117^, ^118^ ^]^ This technique consists of stamping the hydrogel with a negative mold before polymerization has occurred. Otherwise, the hydrogel can be allowed to completely polymerize against a sacrificial mold^[^ ^119^ ^]^ which can then be dissolved to reveal a villous‐patterned gel. In addition to increasing barrier surface area, the surface topography modification would further decrease the distance between fetal vasculature and the maternal compartment of our device.

## 4 Conclusion

Since the placenta is not a static organ, certain drugs, antibodies, bacteria, and viruses may permeate the placental barrier at different rates as a function of trimester and therefore may be deemed safe for limited periods of time. In vitro placental models should therefore be capable of mimicking the various stages of its development to produce translatable permeability data. Here, we have constructed an in vitro placental barrier model from human trophoblast stem cells which has the capacity to model first and third trimester placental function with the careful modulation of trophoblast fusion and vascularization. This work shines a light on the critical effects of perfusable vasculature on molecular clearance within the placenta and, thus, perceived placental barrier permeability. More broadly, this model offers the opportunity to evaluate the safety of certain compounds for use during the different trimesters of pregnancy.

## 5 Experimental Section

### Cell Culture and Media Formulation

Blastocyst‐derived PSCs (RCB‐4940, female) were purchased from Riken BRC Cell Bank (Figure S2, Supporting Information), received at passage 17 and used until passage 23 in all experiments. Cytotrophoblast‐derived PSCs (RCB‐4936, female) were also received from the Riken BRC Cell Bank, however, they were observed to be less robust and proliferative than the blastocyst‐derived cells, and therefore the former were used for all experiments in this work. Cells were cultured in PSC expansion medium^[^ ^15^ ^]^ (PSCM) consisting of Dulbecco's modified Eagle medium (DMEM)/F12 + GlutaMAX supplemented with 2‐mercaptoethanol (0.1 × 10^−3^ m), fetal bovine serum (FBS, 0.2%), penicillin–streptomycin (0.5%), bovine serum albumin (BSA, 0.3%), ITS (insulin, transferrin, selenium) media supplement (1%), L‐ascorbic acid (1.5 µg mL^−1^), EGF (50 ng mL^−1^), CHIR99021 (2 × 10^−6^ m), A83‐01 (0.5 × 10^−6^ m), SB431542 (1 × 10^−6^ m), VPA (0.8 × 10^−3^ m), and Y27632 (5 × 10^−6^ m). Flasks were coated with Col IV (10 µg mL^−1^) in phosphate‐buffered saline (PBS) and incubated for at least 1.5 h at 37 °C before cell plating. Cells were cultured at 37 °C with 5% CO_2_ and media was replaced every 2 days. When cells reached 80% confluency, they were passaged by briefly washing with Dulbecco's phosphate‐buffered saline (DPBS) and dissociating with TrypLE for 10 min, then split at a ratio of 1:4 onto the collagen‐coated flasks. BeWo b30 cells were generously provided by the Raha Lab at McMaster University. They were cultured in DMEM supplemented with FBS (10%) and penicillin–streptomycin (1%). When seeded in regular 384‐well plates, BeWo b30 and PSCs were seeded at a density of 5000 cells well^−1^, whereas on fibrin gel cast in a 384‐well plate, cells were seeded at a density of 24 000 cells well^−1^. PSCs were cultured for 8 days before analysis, whereas BeWo cells were cultured for 4 days before analysis. Primary HUVECs (Cedarlane Labs, CAP‐0001GFP) were cultured in endothelial cell growth medium 2 (ECGM2) and used until passage 5. Primary human lung fibroblasts (FBs, Cedarlane Labs, PCS‐201‐013) were cultured in DMEM supplemented with FBS (10%), penicillin–streptomycin (1%), and 4‐(2‐hydroxyethyl)‐1‐piperazineethanesulfonic acid (1%, Thermo Fisher Scientific, 15630080) and used until passage 5. BeWo cells, HUVECs, and lung fibroblasts were dissociated using trypsin‐ethylenediaminetetraacetic acid solution (0.05%) before being seeded into standard 384‐well plates or IFlowPlate devices.

### PSC and BeWo b30 Syncytial Differentiation

PSCs were differentiated into the syncytiotrophoblast subtype by passaging and immediately seeding in PSC differentiation medium^[^ ^15^ ^]^ (PSCM+D) consisting of DMEM/F12 + GlutaMAX supplemented with 2‐mercaptoethanol (0.1 × 10^−3^ m), penicillin–streptomycin (0.5%), BSA (0.3%), ITS media supplement (1%), Y27632 (2.5 × 10^−6^ m), forskolin (2 × 10^−6^ m), and KSR (4%). PSCs were differentiated for 8 days before analysis and media was changed daily. BeWo b30 cells were differentiated by first culturing them in expansion medium (DMEM supplemented with FBS (10%) and penicillin–streptomycin (1%)) and then supplementing the media with forskolin (50 × 10^−6^ m) and EGF (50 ng mL^−1^) for 2 days, where media was changed daily.^[^ ^22^ ^]^

### Immunostaining

The entire immunostaining procedure of cells cultured on fibrin gels was performed on a programmable rocker (OrganoBiotech, Cat#B001) at 4 °C to allow stains and washes to perfuse through the matrix. Cells were first washed with D‐PBS to remove residual media and were then fixed with paraformaldehyde (4%) in D‐PBS overnight. The next day, cells were washed 3x with D‐PBS for 5 min each and left in D‐PBS overnight to wash off residual paraformaldehyde. Cells were then blocked for at least 1 h with FBS (10%) and triton‐X (0.1%) in D‐PBS. A primary antibody solution was prepared by diluting anti‐e‐cadherin (1:200 dilution) and anti‐hCG (1:50 dilution) in the blocking solution. Samples were incubated in primary antibody solution overnight and washed 3x the next day and washed overnight. A secondary antibody solution consisting of anti‐rabbit (1:1000 dilution), anti‐e‐mouse (1:1000 dilution), DAPI (1:1000 dilution), and FBS (10%) in PBS was applied onto the samples for 2 h at room temperature. After incubation, samples were washed overnight before removing the tissues from the plate and imaging with a confocal microscope (3i Marianas Lightsheet microscope).

### Fusion Percent Quantification

Cells were immunostained for DAPI and e‐cadherin, an epithelial cell adhesion molecule, and fluorescently imaged using a confocal microscope (3i Marianas Lightsheet microscope) to assess cell fusion rates. The total cell number in each image was quantified by counting the DAPI‐stained nuclei. When at least three nuclei were present within a single e‐cadherin boundary, these cells were considered fused. The fusion percent was reported as the fraction of fused cells over the total cell number.

### Histology and IHC

IFlowPlate samples were fixed in 10% formalin at 4 °C for at least 48 h. Tissues were removed from the device and embedded into HistoGel (VWR, CA83009‐992). Samples were allowed to gel at 4 °C overnight and were packed in histology cassettes and submerged in 70% ethanol before being sent to the MIRC histology Core Facility at McMaster University. Tissues were embedded in paraffin wax, sectioned, and stained for H&E, e‐cadherin (Abcam, ab1416), and CD31 (Abcam, ab28364). Histology cross‐sections were imaged in brightfield with a tissue culture microscope (Nikon Eclipse Ts2).

### hCG Secretion Enzyme‐Linked Immunosorbent Assay (ELISA)

PSC and BeWo b30 cell supernatants were analyzed for hCG secretion using a human hCG ELISA kit (Abcam, ab100533) according to manufacturer's instructions. Briefly, 90 µL of media supernatant was collected from samples cultured in either standard 384‐well plates or 384‐well plates casted with fibrin hydrogels. Residual hCG was obtained by washing samples with fresh media (90 µL) and collecting remaining media. 100 µL of each supernatant sample and of antigen standard curve (positive control) were incubated in individual wells of the ELISA array plate overnight at 4 °C with gentle shaking. The next day, wells were incubated at room temperature with gentle shaking with a 1X biotinylated hCG detection antibody (100 µL) for 1 h, then with a 1X HRP‐streptavidin solution (100 µL) for 45 min and finally with 3,3',5,5'‐tetramethylbenzidine (TMB) one‐step substrate reagent (100 µL) for 30 min. Wells were washed four times with 1X wash solution (300 µL each) between each incubation step. Stop solution (50 µL) was added to each well immediately before measuring absorbance values at 450 nm using the Cytation5 microplate reader (Biotek).

### DNA Quantification Assay

PSCs and BeWo b30 cells were cultured in a 384‐well plate either with or without fibrin hydrogel and in their corresponding media. Cells were lysed using a guanidine‐isothiocyanate lysis buffer (100 µL, Purelink RNA Mini kit, Thermo Fisher Scientific, 12183018A) and sonicated in an ice‐bath for 10 s, in pulses. The lysis buffer degraded the fibrin gel matrix which resulted in a homogenous cell lysate. DNA was quantified using the DNAQF kit (Sigma‐Aldrich, DNAQF‐1KT) by following the manufacturer's instructions. Briefly, the DNA standard supplied in the kit was serially diluted in the fluorescent assay buffer to create a standard curve. Next, a bisbenzimide solution (2 µg mL^−1^) was prepared and 200 µL was pipetted into each well of a black‐bottomed 96‐well plate (VWR, 76221–764). 10 µL of each sample and each standard were added to separate bisbenzimide‐filled wells immediately before taking fluorescent reading (360 nm excitation, 460 nm emission) on the Cytation5 microplate reader (Biotek). The DNA standard curve allowed to convert the fluorescent readings to DNA quantities. To obtain a cell number from these values, a calibration curve relating cell number to DNA concentration was created using subcultured PSCs. Cells were lysed with lysis buffer (100 µL) and DNA quantification was performed as described above.

### SEM Imaging

Samples were fixed in paraformaldehyde (PFA, 4%) overnight and removed from IFlowPlate device using a scalpel and tweezers. They were then placed in fresh PFA solution before being sent to the electron microscopy facility (EMF) in the Health Science Center (McMaster University). Once at the EMF, the samples were rinsed 2X in buffer solution, post‐fixed in osmium tetroxide (1%) in phosphate buffer (0.1 m) for 1 h and then dehydrated through a graded ethanol series (50%, 70%, 70%, 95%, 95%, 100%, 100%).  The samples were kept immersed in 100% EtOH, placed into wire baskets, and transferred to the chamber of a Leica EM CPD300 critical point dryer (Leica Mikrosysteme GmbH, Wien, Austria). The chamber was sealed and then flushed 12 times with liquid CO_2_. The CO_2_‐filled chamber was heated to 35 °C and the pressure was increased in chamber to above 1100 psi so that CO_2_ was changed from liquid phase to gaseous phase. The gas was vented slowly from the chamber until atmospheric pressure was reached and the samples were dehydrated without surface tension damage. The dried samples were mounted onto SEM stubs with double‐sided carbon tape. The samples on stubs were then placed in the chamber of an Edwards S150B Sputter Coater and ≈15 nm of gold was deposited onto the stubs.  The samples were viewed in a Tescan Vega II LSU SEM (Tescan USA, PA) operating at 20 kV.

### Transcriptomic Analysis

Microarray sequencing was performed on undifferentiated (PSC Undif) and differentiated PSCs (PSC Dif), as well as differentiated BeWo b30 cells (BeWo Dif). PSCs were seeded in collagen IV‐coated 6‐well plates at a density of 100 000 cells well^−1^ and cultured in 2 mL of either expansion medium or differentiation medium. Media was changed every 2 days and cells were lysed on day 8. Lysates from three wells were combined and used for each replicate to ensure adequate RNA quantities for analysis. BeWo b30 cells were seeded in standard 6‐well plates at a density of 200 000 cells well^−1^ and cultured in expansion medium for the first 2 days. On day 2, the media was switched to differentiation medium, and cells were lysed on day 4. Only a single well's lysate was sufficient for each RNA replicate given high BeWo cell densities at day 4. Samples were lysed and RNA extraction was performed using the Purelink RNA Mini kit (Thermo Fisher Scientific, 12183018A) following the manufacturer's instructions. RNAs were eluted from the spin cartridge using RNase/DNase‐free water (50 µL) and sample quality was assessed using a NanoDrop spectrophotometer (Thermo Fisher Scientific). All samples were ensured to have a 260/280 purity ratios of ≈2.0 and 260/230 purity ratios between 2.0 and 2.2 before being sent to the CRLB‐GMEL facilities for microarray sequencing. Transcriptomic analysis was performed using the Affymetrix Clariom S Assay (human, Thermo Fisher Scientific, 902927) which screened the samples for over 20 000 known human genes. Datasets were imported into the Transcriptomic Analysis Console (TAC) software for analysis. Differentially expressed genes (DEGs) were determined using a one‐way analysis of variance (ANOVA) with *p* < 0.05 and fold‐change criteria of <‐2 for downregulated genes and >2 for upregulated genes. Volcano plots and heat maps were generated using GraphPad Prism.

### Analysis of Public scRNA‐seq Data and Pearson Correlation

To explore the similarity of microarray‐generated transcriptomes to a public single cell RNA‐seq dataset of syncytiotrophoblast cells,^[^ ^27^ ^]^ data (accession E‐MTAB‐6701) were retrieved from the EBI arrayexpress resource. Single cell transcript counts were imported into R v4.2.0 using the readMM() function from the Matrix package v1.5‐1. ENSEMBL gene symbols were then mapped to gene names using the org.Hs.eg.db package v3.15.0, and the genes (*N* = 16461) detected in both the microarray and scRNA‐seq dataset were further analyzed. For each gene, an average syncytiotrophoblast expression level was then calculated as the mean count across all syncytiotrophoblast cells. These values were log‐transformed with an added pseudocount of +1 to avoid division by zero errors. Using the cor() function within R, the Pearson correlation (*r*) coefficient was then computed between the log2 syncytiotrophoblast expression levels of all genes and the microarray signal intensities for the Bewo‐Diff, PSC‐Ctrl, and PSC‐Diff datasets.

### Establishment of Early‐Stage Barrier Model

The early‐stage barrier model consisted of a PSC monolayer cultured on a porous fibrin hydrogel matrix with interconnected adjacent wells. To create this barrier model, our lab's proprietary IFlowPlate model was utilized, which consisted of a 384‐well plate with interconnected well triplets (inlet, center, outlet) and pressure‐adhesive bottoms. Hydrogel casting and cell seeding methodology was adapted from the previous work^[^ ^98^ ^]^ and modified to meet the culture model requirements. IFlowPlate's fabrication was streamlined since the first publication^[^ ^98^ ^]^ and plates were now manufactured in FDA registered, ISO13485 and GMP certified facility (OrganoBiotech, Cat#A001). This device was consisted of three wells interconnected by 200 µm wide by 200 µm tall channels at its base and was compatible with standard 384‐well culture. To set up the culture, first, a fibrinogen pre‐polymer solution (10 mg mL^−1^) was aliquoted into 125 µL quantities. Sterile distilled water (25 µL) was then added to the inlet and outlet wells of the devices being cast. Immediately before casting, thrombin (25 µL, 1 U mL^−1^) was mixed into the fibrinogen solution and 25 µL of this mixture was cast into each center well. Hydrogels were allowed to polymerize at room temperature for 30 min. Water was aspirated from the inlet and outlet wells and appropriate media was added to all three wells. For monolayer cultures, PSCs were seeded onto the hydrogel in the central chamber 1 h after casting (24 000 cells well^−1^) and incubated flat on the rocker overnight for cells to attach. The resulting gel was characterized by an average pore area of 0.54 ± 0.18 µm^2^ and an average Feret diameter of 1.08 ± 0.12 µm (Figure S3A–C, Supporting Information). The next day, the plate was placed on a pre‐programmed rocker (15^o^ tilt, 4 tilts h^−1^), inducing a basolateral media flow rate of 30.0 ± 8.6 µL h^−1^ (Figure S3D,E, Supporting Information), and media was changed daily. All types of media used in the device were supplemented with 20 µg mL^−1^ aprotinin to prevent fibrin degradation.

### Dextran Permeability Assay

Dextran permeability was assessed in IFlowPlate to evaluate the passive diffusion of small molecules through the barrier model. Dextran is often used in culture permeability assays because it is not metabolized by mammalian cells and it has been shown that it can be used to assess size‐dependent paracellular transport,^[^ ^120^, ^121^ ^]^ in other words, transport across the cell barrier via the intercellular space. This assay consisted of adding fluorescently labeled dextran into the central apical chamber of the device, incubating the device on the tilter and taking fluorescent measurements of the adjacent wells at specified timepoints. A 1:1 molar ratio of 65 kDa TRITC‐labeled dextran (0.2 mg mL^−1^) and 4 kDa FITC‐labeled dextran (0.012 mg mL^−1^) was prepared in the appropriate culture medium. For experimental conditions requiring compartmentalized culture, mixed media consisting of a 50:50 ratio of both media types was prepared to eliminate the effect of media fluorescence on dextran fluorescent readouts. Fresh media (90 µL) was added to the inlet and outlet wells of device, and dextran solution (65 µL) was added to the central well to equilibrate the media levels (25 µL gel + 65 µL dextran = 90 µL in center well). A calibration curve was created by serially diluting the dextran solution and adding 90 µL into each well triplet. Fluorescent measurements were acquired using the Cytation5 plate reader and all measurements on the same plot were performed on the same day of culture. Preliminary assays confirmed that there was no fluorescent bleed‐through between TRITC and FITC channels, which would skew experimental results (Figure S4, Supporting Information).

### Poly(I:C) Treatment and Cytokine Analysis

PSCs were cultured on IFlowPlate for 8 days in either expansion medium or differentiation medium. On day 8, media in the central compartment was supplemented with poly(I:C) (10 µg mL^−1^), a viral mimic that was shown to elicit an inflammatory response in many tissues^[^ ^122^, ^123^ ^]^ and, for the placenta specifically, it was used to induce preeclampsia‐like symptoms in animal and explant models.^[^ ^124^, ^125^, ^126^ ^]^ After 24 h, supernatant from the central (apical) compartment and media from the adjacent (basolateral) compartments were collected. Media supernatant was centrifuged at 1000 *g* for 10 min to precipitate any cell debris, and 70 µL of each sample was collected from the top surface of the fluid sample. Samples were stored in −80 °C until they were sent to Eve Technologies for cytokine analysis. Samples were analyzed using the Human Cytokine Pro‐inflammatory Focused 15‐Plex Discovery Assay Array (HDF15) for the following biomarkers: GM‐CSF, IFNγ, IL‐1β, IL‐1RA, IL‐2, IL‐4, IL‐5, IL‐6, IL‐8, IL‐10, IL‐12(p40), IL‐12(p70), IL‐13, MCP‐1, TNFα. Cytokine concentrations were converted to mass by assuming the 90 µL of supernatant in the central well and a combined 180 µL in adjacent wells. Secretion values under the detectable limit of the assay were set to 0.

### Establishment of Late‐Stage Barrier Model

The late‐stage barrier model consisted of a PSC monolayer cultured on a fibrin hydrogel laden with perfusable vasculature. First, a pre‐polymerized hydrogel cell suspension of HUVECs (2.5 M cells mL^−1^) and fibroblasts (0.5 M cells mL^−1^) was prepared and cast in the central well of the IFlowPlate device and polymerized at room temperature for 30 min, similar to the early‐stage model. A coating solution consisting of fibrinogen (1 mg mL^−1^), thrombin (0.1 U mL^−1^), and aprotinin (20 µg mL^−1^) in ECGM2 was prepared. Water was aspirated out of the inlet and outlet wells and replaced with 100 µL of the coating solution. Media (40 µL) was added to the central well and the plate was placed flat in the incubator overnight. The next day the coating solution was replaced with 80 µL of media, the inlet and outlet media were changed and the plate was placed on the rocker. On day 2, the inlet and outlet wells were seeded with an HUVEC cell suspension (110 µL, 1 M cells mL^−1^) and incubated flat overnight. Media was changed on day 3 and the plate was placed on the rocker to encourage endothelial lining of the device channels. PSCs were seeded on the fibrin gel on day 4, similar to the early‐stage model. Once PSCs were seeded in the central compartment, a compartmentalized media culture commenced: PSC media in the central well (90 µL of PSCM or PSCM+D) and endothelial cell media in the inlets and outlets (90 µL of ECGM2). In addition, the plate was placed on the rocker until the end of culture.

### Coculture Media Tolerance Assay

During compartmentalized culture in the IFlowPlate, it was possible for media mixing to occur, especially in early culture when PSCs had not yet formed a tight barrier separating apical and basolateral compartments. A coculture media tolerance assay was performed to determine at what media mixing ratios PSCs could still form a confluent monolayer on fibrin and endothelial cells could self‐assemble into vasculature. To do this, media mixtures consisting of different ratios of PSC expansion (PSCM) media and endothelial cell expansion media (ECGM2) were concocted: 100% PSCM, 75% PSCM/25% ECGM2, 50% PSCM/50% ECGM2, 25% PSCM/75% ECGM2, and 100% ECGM2. PSCs were seeded at a density of 12 500 cells well^−1^ on fibrin gels cast in a 384‐well plate and cultured for 9 days in each media type. Endothelial cells were suspended in the pre‐polymer hydrogel mixture (25 µL) at a density of 1 M cells mL^−1^, cast into a 384‐well plate and cultured for 3 days.

### Vasculature Morphology Quantification

The vasculature from coculture media tolerance assay was characterized using the open‐access AngioTool software.^[^ ^127^ ^]^ The following variables were inputted into the software: high and low threshold values, vessel thicknesses, small particles, fill holes, and scaling factor. These values were set for each image so as to produce the most accurate vessel segmentation and skeletonization. Total vessel area, average vessel diameter, total vessel length, and the number of vascular junctions were outputted from the software.

### Insulin Permeability Assay

To determine insulin permeability across different placental barrier models, PSCM (95 µL) supplemented with human recombinant insulin (1 × 10^−9^ m) was added into the central maternal compartment of the IFlowPlate device. The plates were then incubated on the programmable tilter and the supernatant samples were collected from each compartment after 24 h. An insulin ELISA kit (Sigma‐Aldrich, RAB0327‐1KT) was used to quantify insulin permeation from the maternal to the fetal compartments according to manufacturer's instructions. Briefly, 80 µL of media supernatant was collected from the maternal compartment, and 100 µL from each fetal compartment. Optimal dilution factors for each sample were determined. 100 µL of each diluted supernatant sample and of antigen standard curve (positive control) were then incubated in individual wells of the ELISA array plate overnight at 4 °C with gentle shaking. The next day, wells were incubated at room temperature with gentle shaking with a 1X biotinylated human insulin detection antibody (100 µL) for 1 h, then with 1X HRP‐streptavidin solution (100 µL) for 45 min, and finally with TMB one‐step substrate reagent (100 µL) for 30 min. Wells were washed four times with 1X wash solution (300 µL) between each incubation step. Stop solution (50 µL) was added to each well immediately before measuring absorbance values at 450 nm using the Cytation5 microplate reader (Biotek). Insulin values below the detectable limit of the assay were set to 0. Insulin concentrations were also measured in the apical (maternal) compartment after the 24 h incubation (Figure S5, Supporting Information).

### Statistical Analysis and Plotting

All plots and statistics were performed using either paired *t*‐test or two‐way ANOVAs with Sidak multiple comparison in the GraphPad Prism software with 95% confidence (*α* = 0.05, *p* < 0.05). Normality was tested using Shapiro–Wilk test and equal variance was tested using *F*‐test. Data in all graphs were plotted as means with standard deviation as error bars.

## Conflict of Interest

B.Z. holds equities in OrganoBiotech, Inc. which is commercializing the IFlowPlate Platform used in this work.

## Author Contributions

S.K. performed the experiments, analyzed the results, and prepared the manuscript. A.S. assisted with the SEM imaging and staining. Z.K. and J.A. contributed to RNA extraction. J.A. assisted with image analysis. S.R. supervised the work and edited the manuscript. B.Z. envisioned the concept, supervised the work, and edited the manuscript.

## Supporting information

Supporting Information

## Data Availability

All microarray gene sequencing data will be available on the Gene Expression Omnibus as of October 24, 2023. [GEO accession number: GSE245763]
