# Supplementary material for: Modeling the Progression of Placental Transport from Early‐ to Late‐Stage Pregnancy by Tuning Trophoblast Differentiation and Vascularization
Source: Adv Healthc Mater. 2023 Nov 7;12(32):2301428. doi: 10.1002/adhm.202301428 (PMC11468690; doi:10.1002/adhm.202301428)
Supplement: Supplementary file 1 — Supporting Information [file ADHM-12-2301428-s001.pdf]

ADVANCED HEALTHCARE MATERIALS

Supporting Information

for *Adv. Healthcare Mater.*, DOI 10.1002/adhm.202301428

Modeling the Progression of Placental Transport from Early- to Late-Stage Pregnancy by Tuning Trophoblast Differentiation and Vascularization

Sonya Kouthouridis, Alexander Sotra, Zaim Khan, Justin Alvarado, Sandeep Raha and Boyang Zhang**

September 18th, 2023
adhm.202301428

Supplementary Materials

Modeling the progression of placental transport from early- to late-stage pregnancy by tuning trophoblast differentiation and vascularization

Sonya Kouthouridis[1], Alexander Sotra[2], Zaim Khan[3], Justin Alvarado[3], Sandeep Raha[4,*], Boyang Zhang[1,2,*].

[1] Department of Chemical Engineering, McMaster University, Hamilton, ON, L8S 4L8, Canada
[2] School of Biomedical Engineering, McMaster University, Hamilton, ON, L8S 4L8, Canada
[3] Department of Biochemistry and Biomedical Sciences, McMaster University, Hamilton, ON, L8S 4L8, Canada
[4] Department of Pediatrics, McMaster University, Hamilton, ON, L8S 4L8, Canada

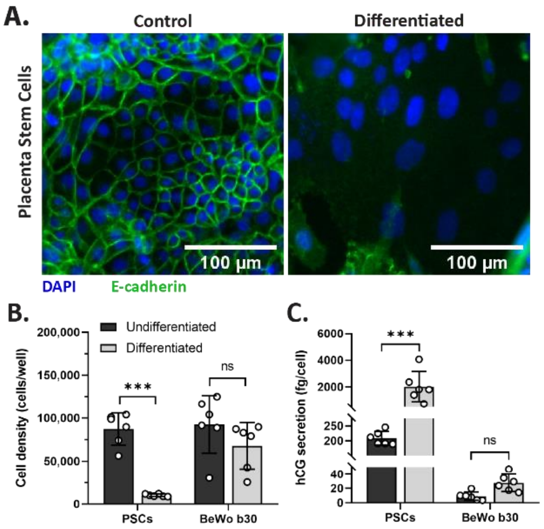

Supplementary Figure 1: Differentiation of PSCs and BeWo b30 cells on tissue culture plastic (TCP). A. PSCs were differentiated in 384-wells for 8 days, fixed and stained for nucleic acids (DAPI, blue) and cell adhesion marker, e-cadherin (green). **B.** Cell density of PSCs and BeWo b30 cells cultured in standard 384-well plates (cells/well) as measured via DNA quantification assay. (one-way ANOVA, N=6, ***p<0.001) **C.** Secretion of hCG over a span of 24 hours. (one-way ANOVA, N=3, ***p<0.001).

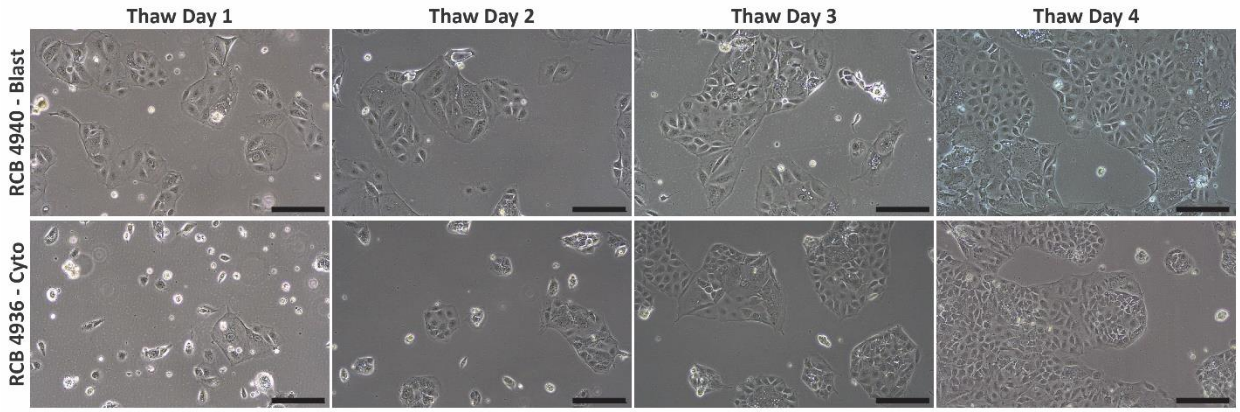

Supplementary Figure 2: Thawing and expansion of PSCs. Phase contrast images of showing the growth of blastocyst- (RCB-4940) and cytotrophoblast-derived (RCB-4936) PSCs in cell culture flasks. (100 um scale bar)

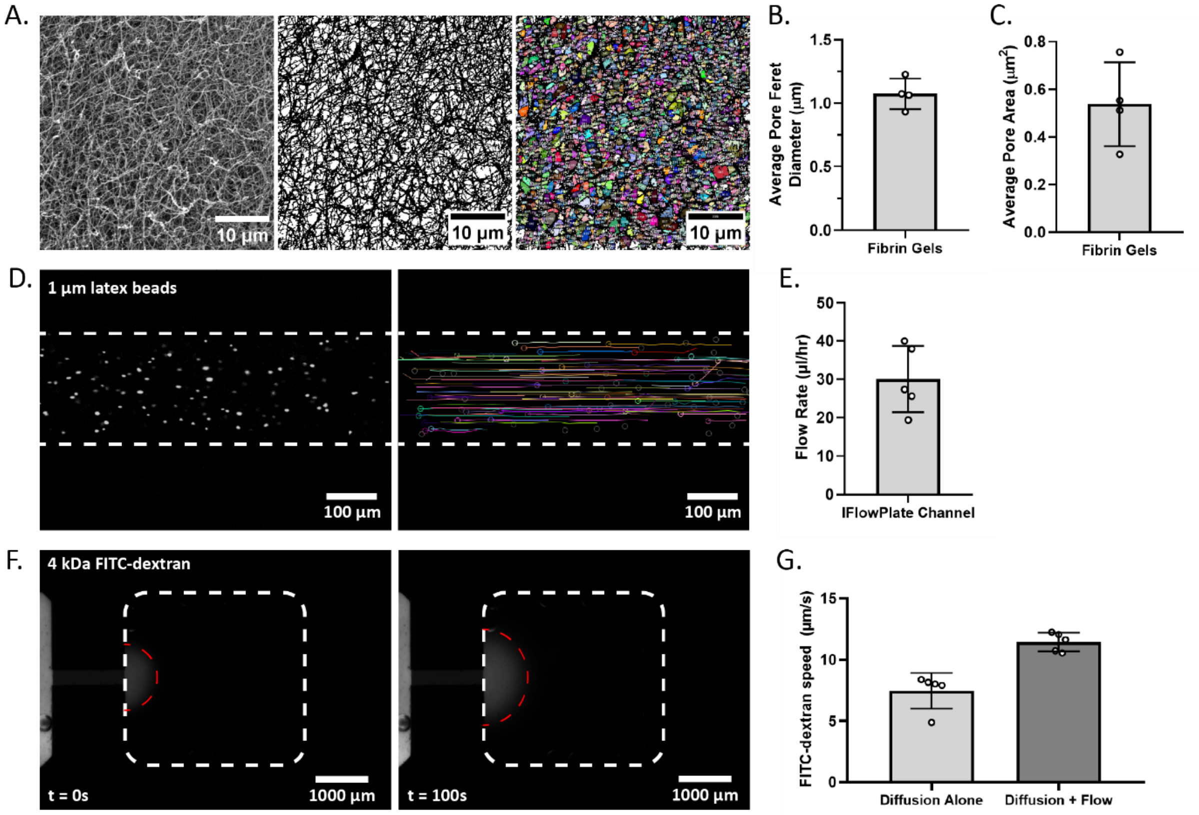

Supplementary Figure 3: Fibrin gel characterization. A. SEM image of fibrin pores and image processing steps including thresholding and pore area measurements. **B.** Average Feret diameter of fibrin gel pores. (N=4) **C.** Average area of fibrin gel pores. (N=4) **D.** 1 µm latex bead particles moving through IFlowPlate channel adjoining its wells and respective Trackmate particle movement analysis tracers. **E.** Flow rate measurements through IFlowPlate channel during pressure-driven flow obtained from 1 µm latex particle movement. **F.** 4 kDa FITC-dextran perfusion through fibrin hydrogel from left IFlowPlate compartment at t=0s and t=100s. **G.** 4 kDa dextran velocity through fibrin gel via diffusion alone and diffusion with pressure-driven flow.

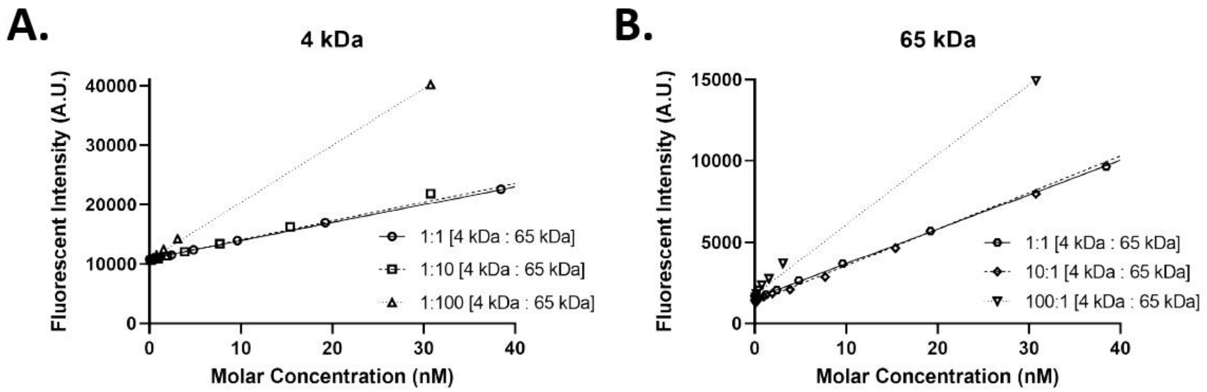

Supplementary Figure 4: Fluorescent standard curves of 4 kDa and 65 kDa dextran when mixing at different ratios. Standard curves are identical when mixing FITC-labelled 4 kDa dextran with TRITC-labelled 65 kDa dextran at ratios of 10:1, 1:1 and 1:10 [4 kDa : 65 kDa], within which is the working range of our assay. Standard deviations are represented as error bars, however they are very small and overlap with the data symbols.

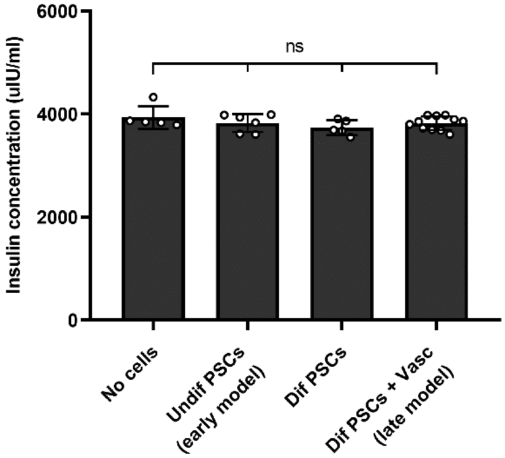

Supplementary Figure 5: Apical concentrations of insulin in late-stage barrier permeability assay. Insulin concentrations in supernatant from apical (maternal) compartments of late-stage placental barrier model. (Two-way ANOVA, N=5 to N=11, *p<0.05). Apical insulin concentrations were statistically similar between all culture conditions, suggesting that insulin binding or degradation by PSCs was not significant.

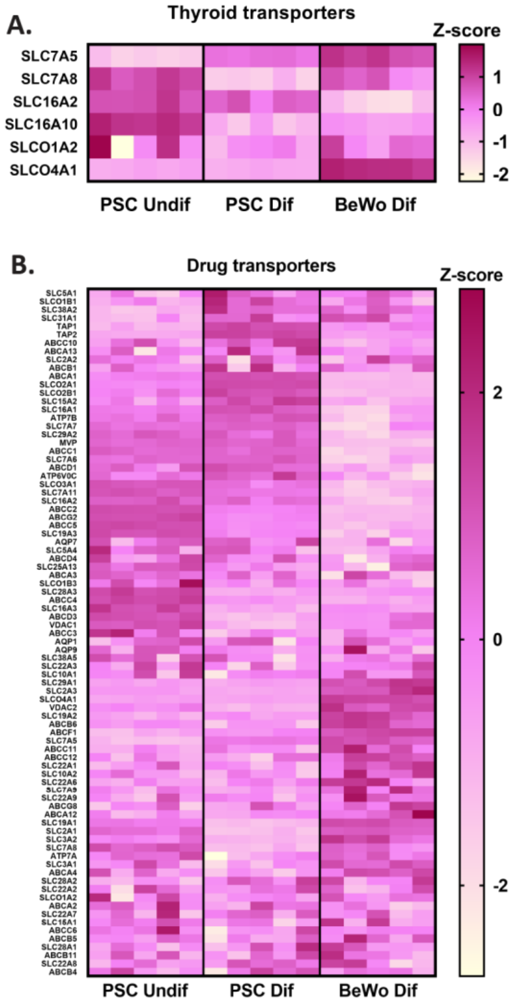

Supplementary Figure 6 Heat map displaying (**A.**) important thyroid transporters[1] and (**B.**) pharmaceutically relevant drug transporters[2] for all three experimental conditions. Gene expression levels were normalized by calculating z-scores. Colours represent scaled expression values where magenta signifies high expression and white, low expression.

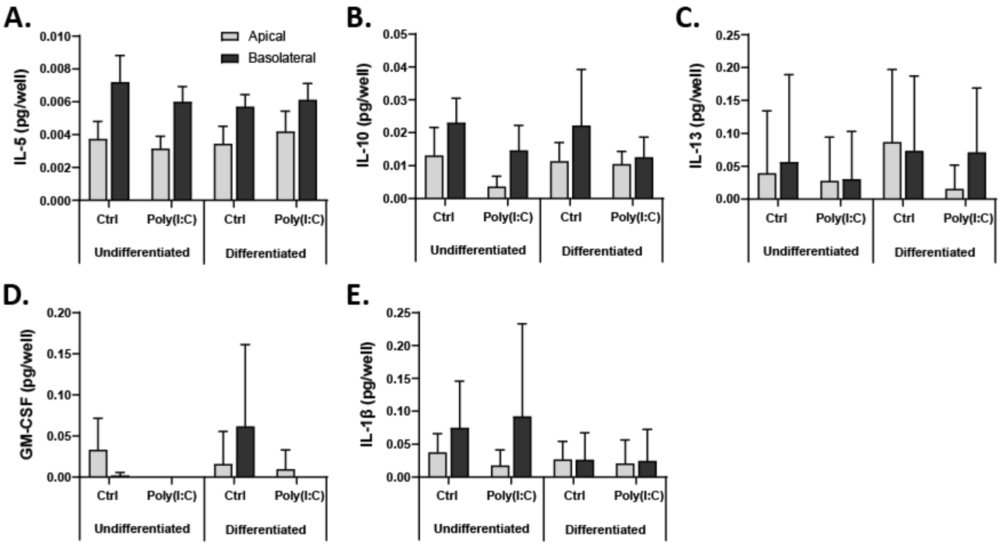

Supplementary Figure 7: Remaining cytokine secretion data in early-placental barrier model. IL-5, IL-10, IL-13 GM-CSF and IL-1β secretion in apical (maternal) and basolateral (fetal) compartments of undifferentiated and differentiated PSCs with and without poly(I:C) treatment. (two-way ANOVA, N=6, *p<0.05, **p<0.01, ***p<0.001)

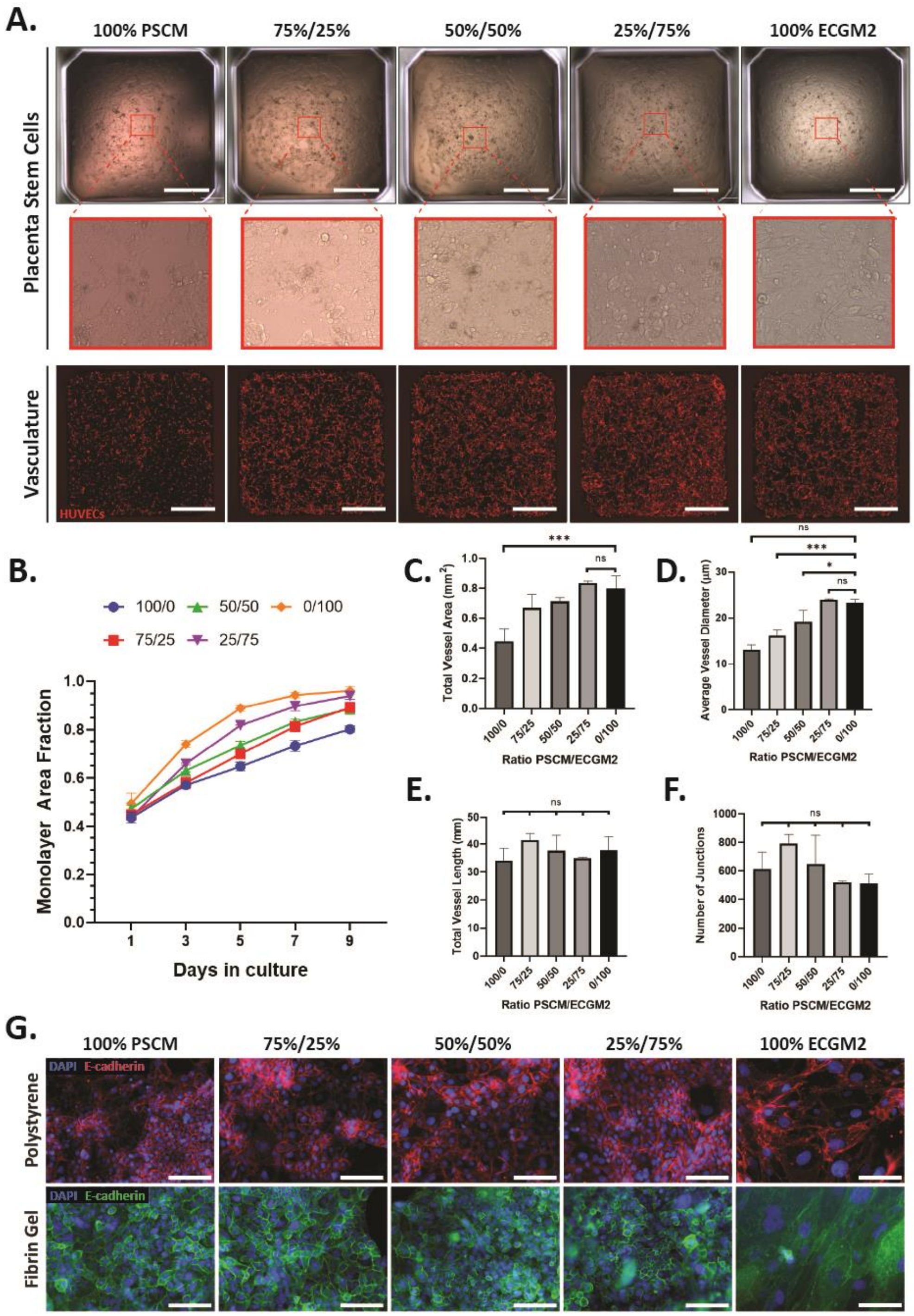

Supplementary Figure 9: Coculture media tolerance assay. A. Brightfield images of PSCs cultured on the surface of fibrin gels for 3 days. Fluorescent images of HUVECs (red) self-assembled within fibrin gels for 3 days. Both cells were cultured with different ratios of PSCM and ECGM2 (100/0, 75/25, 50/50, 25/75 and 0/100, respectively). (1000 um scale bars) **B.** Timelapse of PSC monolayer coverage when culture on fibrin gels. Data presented as averages with standard deviation as error bars. **C.** Total vessel area of HUVECs self-assembled within fibrin gels after 3 days in culture. (One-way ANOVA, N=3, ***p<0.001) **D.** Average vessel diameter. (One-way ANOVA, N=3, *p<0.05, ***p<0.001) **E.** Total vessel length. (One-way ANOVA, N=3, *p<0.05) **F.** Number of vessel junctions (One-way ANOVA, N=3, *p<0.05) **G.** PSCs cultured for 11 days on either 384-well polystyrene tissue culture plates or

fibrin gel with different media ratios of ECGM2 and PSCM. Cells were immunostained for nucleic acids (DAPI, blue) and e-cadherin (red and green). (100 um scale bars)

SUPPLEMENTARY TABLES

Supplementary table 1: Reagents and media supplements

Reagent	Source	Catalog number
DMEM/F12	Thermo Fisher Scientific	10565018
2-mercaptoethanol	Sigma-Aldrich	M3148-25ML
Fetal bovine serum (PBS)	Thermo Fisher Scientific	124840128
Penicillin-streptomycin	Wisent Bioproducts	450-201-EL
Bovine serum albumin (BSA)	Sigma-Aldrich	A9205
ITS liquid media supplement (100x)	Sigma-Aldrich	I3146
L-ascorbic acid	Sigma-Aldrich	A8960
Epidermal growth factor (EGF)	STEMCELL Technologies	78006.1
CHIR99021	STEMCELL Technologies	72054
A83-01	STEMCELL Technologies	72022
SB431542	STEMCELL Technologies	72234
Valproic acid (VPA)	STEMCELL Technologies	72292
Y27632	STEMCELL Technologies	72304
Collagen IV	Sigma-Aldrich	C7521-5MG
Forskolin	STEMCELL Technologies	72112
Knockout serum replacement (KSR)	Thermo Fisher Scientific	10-828-028
TrypLE	Thermo Fisher Scientific	12-605-010
Trypsin	Thermo Fisher Scientific	25300120
D-PBS	Thermo Fisher Scientific	14190144
Endothelial growth medium (ECGM2)	Sigma-Aldrich	C-22111
DMEM	Thermo Fisher Scientific	11995-065
RNase/DNase-free distilled water	Thermo Fisher Scientific	10977-023
Fibrinogen	Sigma-Aldrich	F3879-5G
thrombin	Sigma-Aldrich	T6884-100UN
Bovine serum albumin (BSA)	Sigma-Aldrich	A9418-100G
paraformaldehyde	Electron microscopy sciences	EMS 15710-S
Fetal bovine serum, qualified, heat inactivated, Canada (FBS)	Thermo Fisher Scientific	12484028
Triton-X	Sigma-Aldrich	T8787-50ML
65 kDa TRITC dextran	Sigma-Aldrich	T1162-100MG
4 kDa FITC dextran	Sigma-Aldrich	46944-100MG-F
Human recombinant insulin	Sigma-Aldrich	91077C-100MG

Supplementary Table 2: Antibodies

Reagent	Source	Catalog number
Anti-e-cadherin (IF and IHC)	Abcam	ab1416
DAPI	Sigma-Aldrich	MBD0015-1ML
Anti-rabbit IgG (CF594)	Sigma-Aldrich	SAB4600107-250UL
Anti-mouse IgG (CF594)	Sigma-Aldrich	SAB4600105-250UL
Anti-mouse IgG (FITC)	Sigma-Aldrich	F0257-1ML
Anti-hCG	Sigma-Aldrich	SAB4500168-100UG
Anti-CD31	Abcam	ab28364

SUPPLEMENTARY METHODS

Diffusion and flow velocity though IFlowPlate
25 µl of a 4 kDa FITC-labelled dextran in PBS solution was added to the left compartment of a cell-free IFlowPlate device with no liquid in the center and right compartments. Dextran movement was then monitored via fluorescent imaging (Cytation5, BioTek) for 100 seconds. Timelapse images were imported into imageJ, thresholded and half-ellipses were manually overlayed onto the areas with dextran. Changes in the horizontal axis of the ellipse were measured and the velocity of dextran movement was determined. The effects of diffusion were accounted for by repeating this experiment, however instead of no liquid in the right chamber, 25 µL of PBS was added to equalize the pressure in all compartments. Dextran movement was then image at t=0s and t=100s using a plate reader (Cytation5, BioTek). Dextran movement from diffusion was subtracted from the previous measurements to obtain a final flow velocity. These values were then compared to flow velocity of 1 µm particles perfused through IFlowPlate vasculature acquired by the author of our previous publication[3], which were originally used for their shear stress calculations.

Pore size analysis of fibrin
Four fibrin gels were extracted from the IFlowPlate device, dehydrated and SEM imaged at 5kx to determine their porosity. Images were imported into the ImageJ software, cropped, and their brightness and contrast were automatically optimized according to the software's algorithm. The 8-bit grayscale images were then thresholded between 0 and 80 and small background dots were eliminated using the *fill holes* command. The resulting binary image adequately represented the pores on the outmost surface of the gel (**Supplementary Figure 3A**), which were characterized using the *Analyze Particles* function (pixel range 50-5000 pixels, circularity 0-1, exclude on edges).

Flow rate through IFlowPlate
The flow rate of media through the IFlowPlate gel was measured by perfusing 1 µm FITC-labelled latex beads (L1030, 2.5% solids, Sigma-Aldrich) throughout the device. 25 µL of a 1:800 dilution solution of latex beads in PBS were added into the left compartment, with no fluid in the center of right wells, to simulate the 15° tilt angle of the rocker. Timelapse videos of the beads moving through the channel were recorded using a confocal (3i Marianas Lightsheet microscope) at an average timelapse interval of 100 ms. These images were then imported into imageJ and bead movement was analyzed using the TrackMate plugin[4] with the following settings: 10 µm estimated object diameter, 200 quality threshold, 1615 initial thresholding, 60 µm initial search radius, 20 µm search radius, 1 frame max frame gap, >4.55 number of spots in track, <1.0 mean directional change rate, >0.9 linearity of forward progression. Average particle velocity was obtained and, knowing the IFlowPlate square channel dimensions were 200 x 200 µm^2, we were able to calculate the flow rate of media perfusing through the gel.

SUPPLEMENTARY BIBLIOGRAPHY

1. Loubière, L. S. *et al.* Expression and Function of Thyroid Hormone Transporters in the Microvillous Plasma Membrane of Human Term Placental Syncytiotrophoblast. *Endocrinology* **153**, 6126–6135 (2012).
2. Berveiller, P. *et al.* Drug transporter expression during in vitro differentiation of first-trimester and term human villous trophoblasts. *Placenta* **36**, 93–96 (2015).
3. Rajasekar, S. *et al.* IFlowPlate—A Customized 384-Well Plate for the Culture of Perfusable Vascularized Colon Organoids. *Advanced Materials* **n/a**, 2002974 (2020).
4. Ershov, D. *et al.* TrackMate 7: integrating state-of-the-art segmentation algorithms into tracking pipelines. *Nat Methods* **19**, 829–832 (2022).
